# Effect of Velocity Alignment on the Packing of Active Particles

**DOI:** 10.3390/mi17080884

**Published:** 2026-07-24

**Authors:** Jigarkumar Modi, Ruizhi Jin, Kejun Dong, Gu Fang

**Affiliations:** School of Engineering, Western Sydney University, Sydney 2747, Australiag.fang@westernsydney.edu.au (G.F.)

**Keywords:** active particles, collective behaviour, local interaction rules, velocity alignment, confined environment, packing behaviour

## Abstract

The dynamics of active particles are increasingly being leveraged to design and control micro-robotic swarms. Local interactions play a crucial role in the phase transitions of active particles; how the combined effects of alignment, short-range repulsion, and boundary interactions regulate their packing structure and collective order with different confinement scales remains less systematically explored. In this study, we investigate the packing of active particles within a confined region, focusing on the role of local interaction rules in shaping both the packing structure and the polar order parameter. The effects of key controlling variables related to local interaction rules, including interaction radius, repulsion radius, confined boundary radius, and noise strength, are numerically studied. Specifically, by comparing systems with and without velocity–alignment interactions, we reveal the role of alignment in dictating both structural and dynamical properties of the ensemble. To quantify the packing structure, we employ Voronoi tessellation to evaluate both local and global packing densities. The results show that strong confinement induces a jammed state in which alignment effects are suppressed, resulting in high global packing density and low polar order, regardless of the noise amplitude. Upon increasing the boundary radius beyond a critical threshold, the system unjams, enabling alignment interactions to significantly enhance both the polar order parameter and packing density. Interestingly, the relationship between global packing density and micro-structural parameters, such as coordination number and Voronoi tessellation metrics, is similar in the systems with and without alignment. Our results demonstrate that collective packing and phase behaviour of active matter are governed by the nontrivial interplay between alignment, confinement, and noise, with alignment interactions driving the transition from disordered to ordered states as geometric constraints are relaxed, offering critical insights for the design of targeted micro-robotic swarms and active microfluidic sorting systems.

## 1. Introduction

Active matter has gained increasing research attention in recent years due to its fascinating collective behaviours and broad relevance to systems ranging from soft matter physics to biological systems [1], from artificial [2,3] to natural systems [4,5,6], and spanning an enormous range of length scales from the cell cytoskeleton [4] to the bacterial colonies [5], tissues [7], and animal groups [8]. Active matter encompasses systems in which individual entities can consume energy from their environment and drive their own motion [9,10]. With the “active” feature, these systems exhibit more complex and often counterintuitive phenomena that are not observed in equilibrium statistical mechanics [11], such as spontaneous ordering, pattern formation, and collective motion.

Active matter systems are often modelled by the SSP (Self-Propelled Particle) model, in which each agent is self-propelled and follows local interaction rules. The classical Vicsek model [12] is an early example in which individuals, such as flocks of birds or schools of fish, are idealised as point-like particles that align their velocities with the average direction of their neighbours. The simple alignment interaction can generate collective motion and a global “polar order” in active systems. Subsequent SPP models have incorporated additional mechanisms, including short-range repulsion [13], adaptive speed and repulsion to prevent collision [14], perception cone [15], and a random connection [16,17]. In dense systems of purely repulsive active Brownian particles, aggregation can also occur through motility-induced phase separation, where particles accumulate in crowded regions because their effective motility decreases with increasing local density [18]. These studies demonstrate that local interaction rules can strongly regulate spatial organisation, cluster formation and phase transitions in active matter [11,19,20].

Confinement provides another control mechanism because boundary geometry can reorganise particle orientation, motion and density. For self-aligning soft active discs in a circular trap, Rana et al. showed that trap steepness controls the transition from a polar boundary layer to a compact cluster with hexagonal spatial order; velocity alignment also slows the decay of this spatial order once the trap is removed [21]. Hennes et al. found that hydrodynamically interacting active Brownian particles in a harmonic trap can form a self-induced polar pump, while Caprini et al. showed that activity persistence in an anharmonic trap can produce delocalisation and interaction-assisted freezing [22,23]. More complex dynamic states arise when chirality is included: Lei et al. reported phase separation, living clusters, vortices, swarms and oscillations in confined chiral Janus particles with anisotropic interactions [24] and Negi et al. showed that chirality combined with polar particle alignment and nematic wall alignment can generate edge currents, flocks, ordered oscillations, sustained spirals, counter-rotating currents and travelling bands [25].

These studies establish that confinement, alignment, activity and chirality can generate rich dynamic phases. However, the resulting packing efficiency and microstructural organisation of confined active particles have received less systematic attention, and the effect of velocity alignment has not been discussed. The combined effects of the different local interaction results on global packing density, local Voronoi density, coordination number and polar order therefore remain comparatively underexplored. This gap is important for confined robotic swarms, microfluidic active particle sorting and other systems where particles must not only move collectively but also occupy limited space efficiently [26,27].

By comparison, particle packing in “passive” granular particles has been extensively studied due to its fundamental importance [28,29,30,31], offering insights applicable to active particle systems. The understanding of granular packings has been gradually deepened from the macroscopic to the microscopic scale, particularly using microscopic mechanistic computer models, such as the discrete element method [32]. Furthermore, it was found as early as the 1960s that packing density, or porosity [18,19], is a simple and effective macroscopic parameter to characterise packing structure, whereas understanding its microscopic origins has proven more challenging. Researchers have gained insight into the microscopic structure of packings by analysing local structures using different structural parameters, such as coordination number and Voronoi tessellation [20]. The studies on the relationship between packing density and microstructural parameters revealed how particle–particle interactions lead to jamming and order–disorder transitions [15,16,18,19,20,21,22]. In addition, the boundary of a granular system is also found to be important in shaping the local structures near the boundary and even inducing the order transition of the whole system [33,34].

In active matter systems, the packing behaviour of active particles governed by combined interaction rules under different confinement scales, where boundaries introduce additional complexities that affect local and global dynamics, has received comparatively limited attention. This is particularly relevant to more realistic active systems, such as bacterial colonies in microfluidic channels and robotic swarms operating in constrained space environments [11]. Some studies suggest interesting links between the behaviours of active particles and “passive” granular particles [35]. At higher densities, the system can sometimes lead to packing fractions close to the random close packing (RCP) limit [36]. While it is known that active particles can form dense clusters and experience jamming, fewer studies have systematically applied the methods developed for granular packings to analyse the structure of active particle systems. These methods have provided valuable insights into the packing and statistical behaviour of granular materials and could similarly prove useful in studying active particle packings. Furthermore, such structural analyses could offer a new perspective on active particle systems, drawing on concepts familiar from granular physics, and help clarify how local interaction rules for active particles lead to distinct behaviours compared to “passive” particles.

In this paper, we present a detailed study of the packing behaviour and structure of self-propelled particles confined within a circular boundary subject to repulsion and alignment interactions. With a particular focus on the role of local alignment interactions in shaping packing configurations, we perform numerical simulations with and without these interactions. We analyse not only packing structures using Voronoi tessellations and coordination number but also changes in global and local polar order numbers to uncover the mechanisms driving emergent organisation under confinement, distinguishing the effects of local interactions from geometry (passive) and alignment (active). Our findings demonstrate that different scaled repulsion radii and confinement boundaries establish a baseline, geometry-induced packing, whereas local alignment reorganises the particle-neighbour network and couples this structural reorganisation to collective polar motion. Consequently, aligned and non-aligned systems exhibit distinct coordination number statistics and local and global polar-order profiles under otherwise identical confinement conditions. These results identify local alignment as the active mechanism linking microscopic packing topology to macroscopic collective order and may inform the design and control of confined active systems, including robotic swarms [37,38].

## 2. Numerical Model

**Figure 1:**
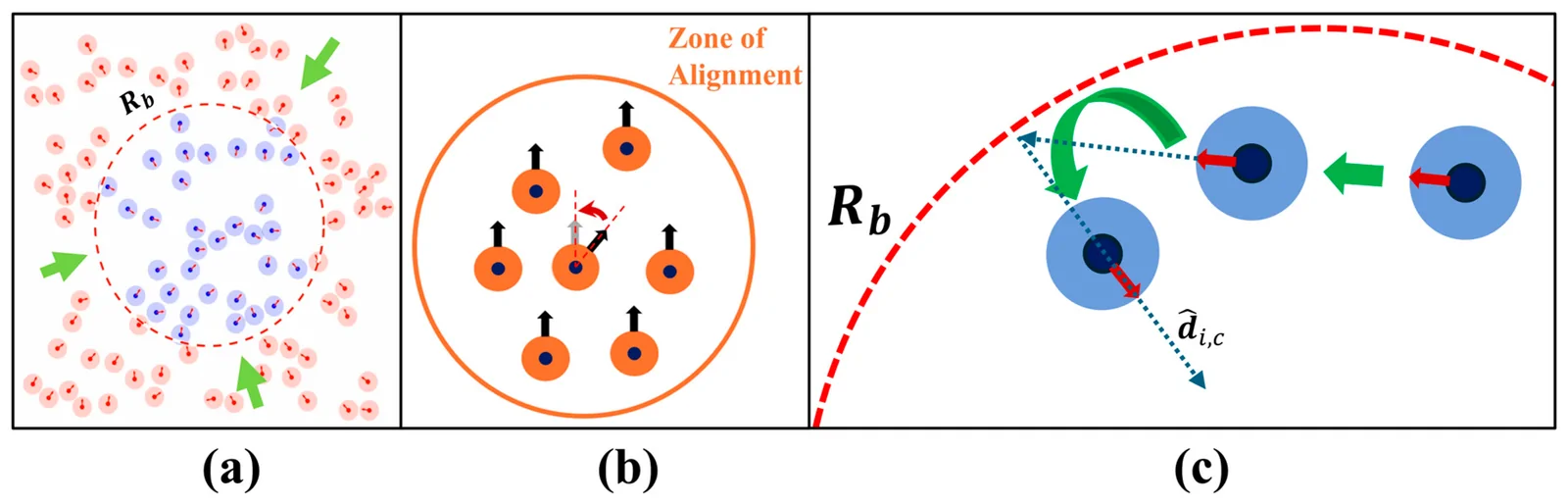
Schematics of the system: (**a**) active particles outside the circular boundary moving with constant speed v towards the circular boundary due to boundary attraction; (**b**) each particle interacts with neighbouring particles within a radius; (**c**) particles inside the boundary are directed toward the centre of the circular boundary when trying to escape.

In this study, we simulate the packing of active particles confined within a circular boundary. Our system consists of *N* active particles; each particle is moving with constant velocity in a simulation domain of dimensions $L_{x}\times L_{y}$. The velocity direction of each particle is updated based on local interactions, including alignment, repulsion, and boundary interaction. Each particle interacts with the neighbouring particles and the boundary within a specified interaction radius, as shown in Figure 1.

Initially, all particles are randomly generated in the simulation domain, with moving directions also randomly generated in [0, 2π]. The moving direction vector of the particle i is denoted as $\theta_{i}=\left(\cos\theta_{i},\sin\theta_{i}\right).$ Then, particles are driven by the boundary interaction rule and particle interaction rules, which are detailed below. Note that all particles maintain the same velocity magnitude, and the interaction rules only change their moving directions.

### 2.1. Particle–Particle Interaction

Here, repulsion and alignment are considered in particle–particle interactions. This concept is adapted from the social force model, which was used in pedestrian dynamics and collision avoidance using repulsive interactions [39]. The repulsion interaction is given by

(1) $$\theta_{r,i}=-\sum_{j\in N_{r}}k_{r}\cdot {\left({R_{r}-d}_{ij}\right)}^{2}\cdot {\hat{d}}_{ij,}$$

where $\theta_{r,\;i}$ represents the velocity direction resulting from the repulsion interaction, ${\hat{d}}_{ij}$ is the direction vector from particle i to particle j, and $k_{r}$ is the repulsion constant, which controls the strength of the repulsion; the summation is over all the particles with $d_{ij}<R_{r}$, and $R_{r}$ and $N_{r}$ are the repulsion radius and the number of particles for repulsion, respectively. Here, we set a high value for the repulsion constant $\left(k_{r}=10^{6}\right)$ to ensure that particles do not overlap, effectively creating a strong barrier that prevents particles from encroaching upon each other [40]. This strong repulsive interaction results in behaviour like a solid wall, maintaining clear separation between individual particles.

We also introduce an alignment interaction, similar to that used in the Vicsek model [12]. The alignment interaction causes particles to align their directions with those of neighbouring particles, thereby achieving collective orientation and synchronised motion. The alignment interaction of the particle i is given by

(2) $$\theta_{a,i}=k_{a}\cdot \frac{\sum_{j\in N_{a}}\theta_{j}}{{\left\|\sum_{j\in N_{a}}\theta_{j}\right\|}^{\prime }}$$

where $\theta_{a,\;i}$ represents the velocity direction resulting from the alignment interaction, $k_{a}$ is the alignment constant ($k_{a}$ = 1 or 0), and the summation is over all the particles with $d_{ij}<R$, and including particle i itself, $N_{a}$ is the number of particles in this set, and R is the interaction radius for alignment.

### 2.2. Particle–Boundary Interaction

In order to allow particle packing within a confined boundary, a circular region with the centre at $(x_{c},y_{c}$) and a radius of $R_{b}$ is considered. When the particle i is outside the circle, it is attracted towards the circle, which is similar to the target-oriented driving interaction [41]. A random direction vector to the target point (${\hat{d}}_{i,t}$) is assigned to each particle for uniform distribution within $R_{b}$. When particle i is already inside the circle and attempts to escape from the boundary, a strong repulsion is applied [42] if the distance between particle i and the circular boundary is less than the repulsive radius ($R_{r}$). That is, particles are allowed a single crossing of the boundary to enter inside. After entering, particles are confined within the boundary and are not permitted to exit the domain. The boundary interaction on the particle i is given by

(3) $$\theta_{b,i}=\left\{\begin{array}{cc} k_{b}\cdot {\hat{d}}_{i,t}, & if\;d_{i,b}>R_{b}\\ k_{r}\cdot d_{i,b}{\cdot \hat{d}}_{i,c}, & if\;d_{i,b}<R_{r} \end{array}\right.$$

where $k_{b}$ is a boundary constant ($k_{b}$ = 3). Here, $k_{r}$ is the repulsion constant, $d_{i,b}$ is the distance between the particle i and the circular boundary, ${\hat{d}}_{i,t}$ is the direction vector from particle i to a random target point inside the circle, and ${\hat{d}}_{i,c}$ is the velocity direction towards the circle centre. Here, we also set a high value for the repulsion constant ($k_{r}$ = 10^6^) to ensure that particles do not penetrate the boundary [40]. Importantly, the circular boundary acts only as a geometric confinement; particles interact with each other when their interparticle interactions are determined solely by distance-based rules. All the interactions are summed and normalised to determine the velocity direction of the particle in the next time step, which is given as

(4) $$\theta_{i}(t+dt)=\frac{\theta_{r,i}+\theta_{a,i}+\theta_{b,i}}{\left\|\theta_{r,i}+\theta_{a,i}+\theta_{b,i}\right\|}$$

In addition, a stochastic angular perturbation ($\varepsilon_{i}(t)$) is applied to the direction of particle i. The perturbation is randomly drawn from a uniform distribution over the interval $\left[-\eta ,\eta \right]$, where η is the noise strength and determines the magnitude of the angular perturbation. The calculated direction $\theta_{i}\left(t+dt\right)$ is rotated by the angle $\varepsilon_{i}(t)$. The resulting direction is given by

(5) $$\theta_{i}^{\prime }\;\left(t+dt\right)=\left(\begin{array}{cc} \cos\varepsilon_{i}(t) & -\sin\varepsilon_{i}(t)\\ \sin\varepsilon_{i}(t) & \cos\varepsilon_{i}(t) \end{array}\right)\cdot \theta_{i}\left(t+dt\right),\;\;\varepsilon_{i}\left(t\right)\sim U\left[-\eta ,\eta \right]$$

**Figure 2:**
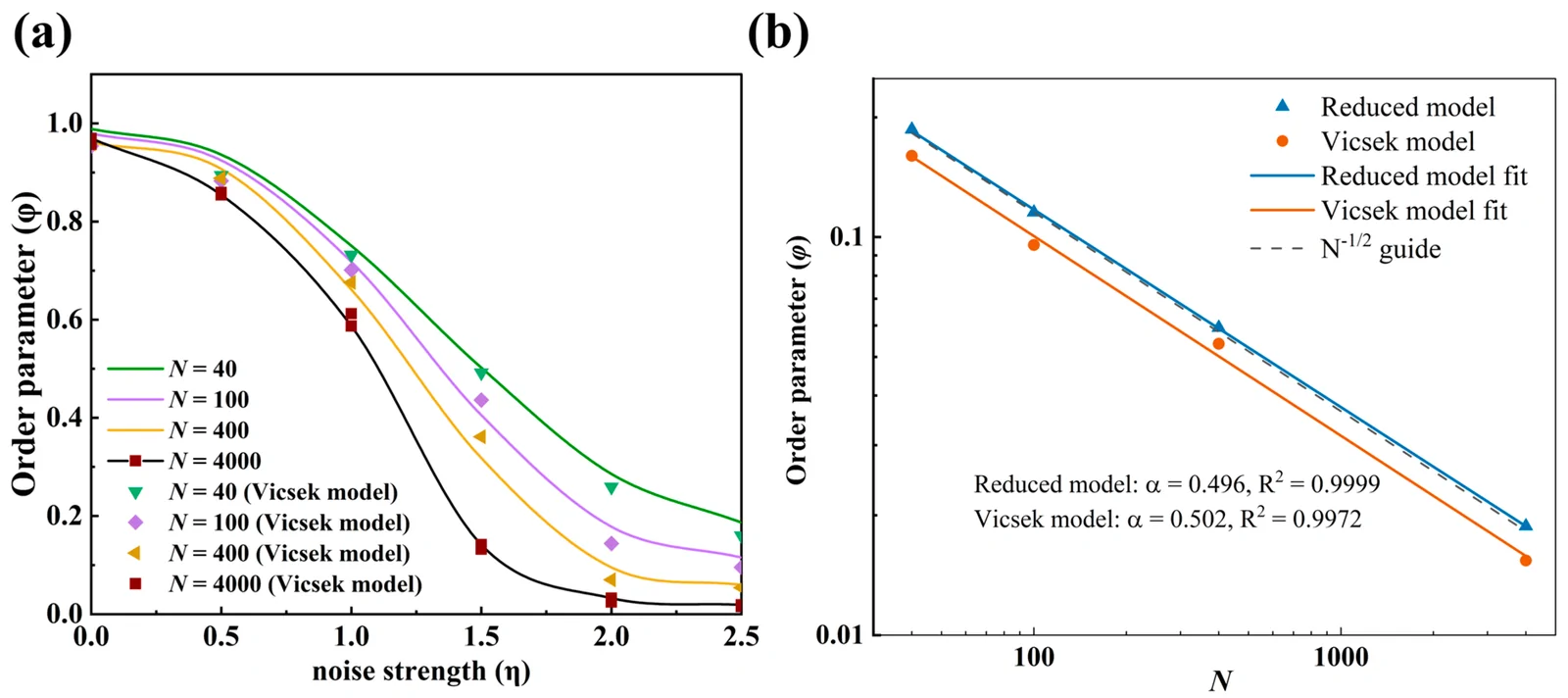
Verification of the Vicsek model limit. (**a**) Order parameter as a function of noise strength for N = 40, 100, 400 and 4000. Solid lines represent the reduced present model, while symbols represent the Vicsek model [12]. (**b**) Finite-size dependence of the high-noise residual order at η = 2.5. Power-law fits of the form $\bar{\varphi }\left(N\right)=AN^{-\alpha }$. The dashed line indicates an $N^{-1/2}$ reference dependence. Simulations were performed using periodic square domains with side lengths L = 3.16, 5, and 10, corresponding to number density ρ = 4. The interaction radius R = 1, particle speed $v_{0}$ = 0.1, and time step ∆t = 1 were fixed.

This noise ($\varepsilon_{i}(t)$) introduces randomness into the particle’s movement, thereby mimicking the stochastic fluctuations observed in real-world systems. The position of the particle for the next time step is given by

(6) $$x_{i}(t+dt)=x_{i}(t)+v\cdot dt\cdot \theta_{i}^{\prime }\;\left(t+dt\right),$$

where $x_{i}(t+dt)$ and $x_{i}(t)$ represent the position of a particle i at time t+dt and *t*, respectively. When only the alignment interaction is retained, the present model is reduced to the Vicsek model. To obtain this limit, we remove the particle–particle repulsive interaction and the particle–boundary interaction, eliminate boundary confinement, and impose periodic boundary conditions. Under these conditions, only the local alignment interaction and angular noise are retained. The reduced present model was compared with the Vicsek model results [12] using identical particle numbers, number density, interaction radius, particle speed, time step, noise convention, neighbourhood definition, and synchronous-update procedure. The global order parameter was denoted by φ and calculated as $\varphi =\frac{1}{N}\left|\sum_{i=1}^{N}\theta_{i}\right|$, where N is the total number of particles and $\theta_{i}$ is the unit orientation vector of particle i. Figure 2a shows close agreement between the reduced model and the Vicsek model for the investigated order parameter over the range of noise strengths. Across the simulated combinations of particle number and noise strength, the root mean square difference between the order parameters was 0.0241. To examine the system-size dependence in the high-noise regime, the results with η = 2.5 were fitted to $\bar{\varphi }\left(N\right)=AN^{-\alpha }$, where the exponent α characterises how rapidly the residual order decreases with increasing system size (see Figure 2b). The fitted exponents were α = 0.496 for the reduced present model and α = 0.502 for the Vicsek model. Both values are close to the expected value α = 1/2, indicating that the residual high-noise order is consistent with an approximate $N^{-1/2}$ dependence over the investigated range N = 40–4000. The comparison verifies that the numerical implementation recovers the Vicsek model limit when the additional interactions are removed.

A simulation runs for 1000 timesteps, with a time step dt=1. The system normally reached a steady state after 250 timesteps, which will be shown in the results section.

Notably, when noise strength was very high and the relaxation time was much longer, we extended the simulation time up to 5000 timesteps to ensure that steady-state behaviour was properly captured. The simulation parameters are the size of the domain, the radius of the circular boundary, particle velocity, noise strength, the radius of interaction for alignment, and the radius of repulsion applied to the system (see Table 1). Distances are reported in simulation length units, time in simulation time units, and speed in simulation length units per simulation time unit. A series of controlled simulations is conducted with varying noise strength levels, boundary radii, and repulsion radii to investigate the effects on system stability and pattern formation.

**Figure 3:**
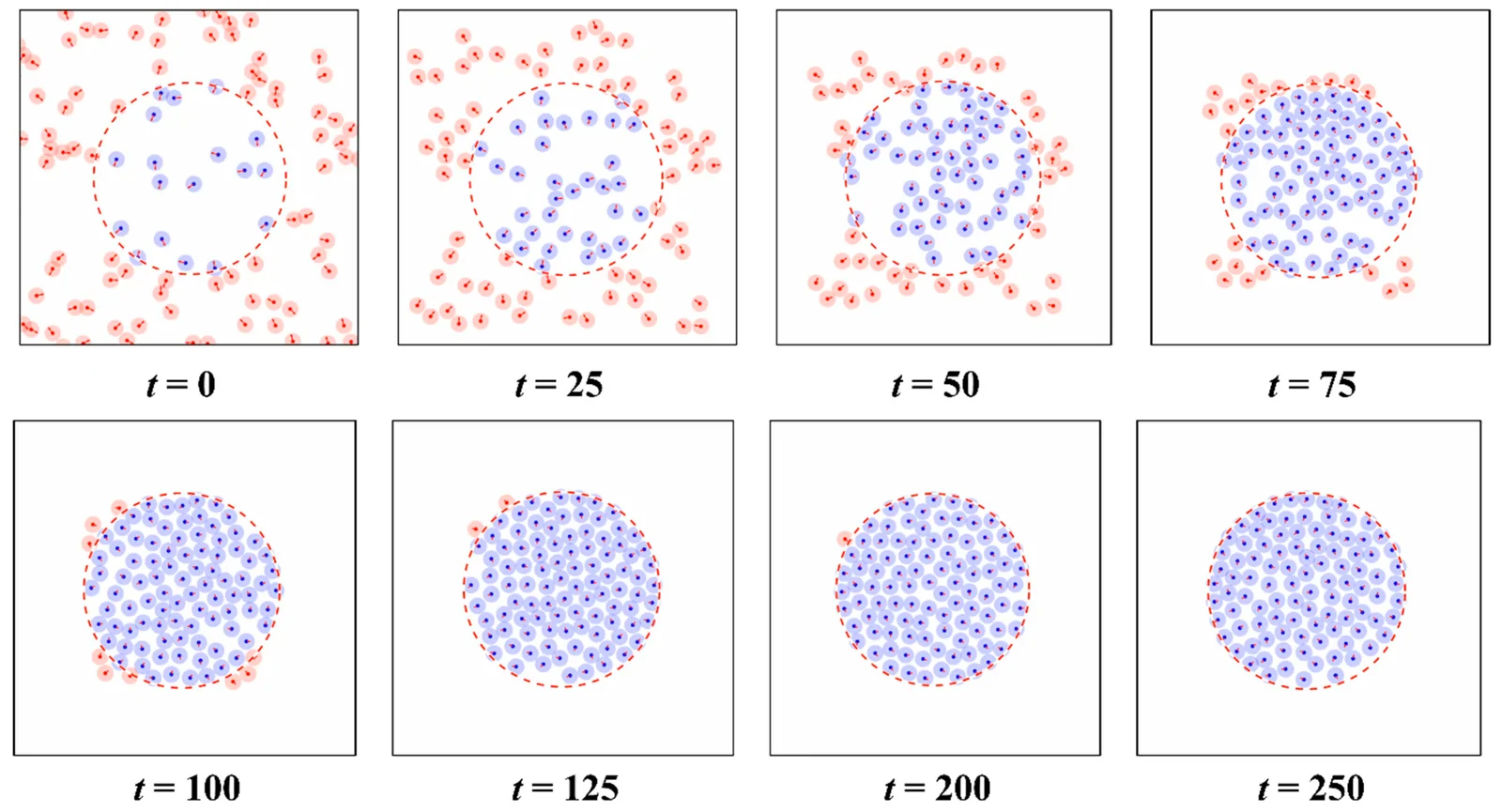
Snapshots of a simulated packing process of active particles. The dashed circle denotes the confining boundary ($R_{b}$), while the small circles represent active particles of radius ($R_{r}$). Particles outside the boundary are shown in red and are attracted towards the confined region, whereas particles inside the boundary are shown in blue and remain constrained by it. The same visual convention is used throughout the other figures.

Figure 3 shows the simulated packing process of active particles into the confined boundary. The blue and red circles are the particles inside and outside the boundary, respectively. At the initial time step (*t* = 0), the particles are randomly distributed and subsequently move towards the boundary. Once the particles enter the boundary, they undergo positional rearrangement within this confined region, leading to the formation of a densely packed structure, which can be observed in Figure 3 at *t* = 250. Once particles enter the circular boundary $R_{b}$, this boundary functions as a repulsive wall (see Supplementary Videos S1–S4). This wall prevents particles from escaping the boundary.

### 2.3. Local and Global Packing Density Calculated by Voronoi Tessellation

In 2D granular packings, packing density is defined as the total area of the particles divided by the area of the whole packing. Here, since particles have a repulsion radius of $R_{r}$, the area of the particle is calculated as $\pi R_{r}^{2}$. Because of the boundary interaction rule, particles will gradually be packed in the boundary, and the packing density will increase over time. However, because active particles are always moving, it will be difficult to measure the entire area of the packing using the static boundary. Here, we use the Voronoi tessellation method to dynamically capture the packing space and calculate the local and global packing density. Voronoi diagrams provide a natural way to quantify local density variations by associating each particle with a distinct region of space. This method allows for a systematic and non-arbitrary determination of the local density, which has been widely used in establishing theoretical models for granular packings [43,44,45,46]. Here, we apply the methodology to active particle packings to gain insights into the local structures of active particles and also establish a direct, quantitative comparison between active and passive (granular) particle systems.

**Figure 4:**
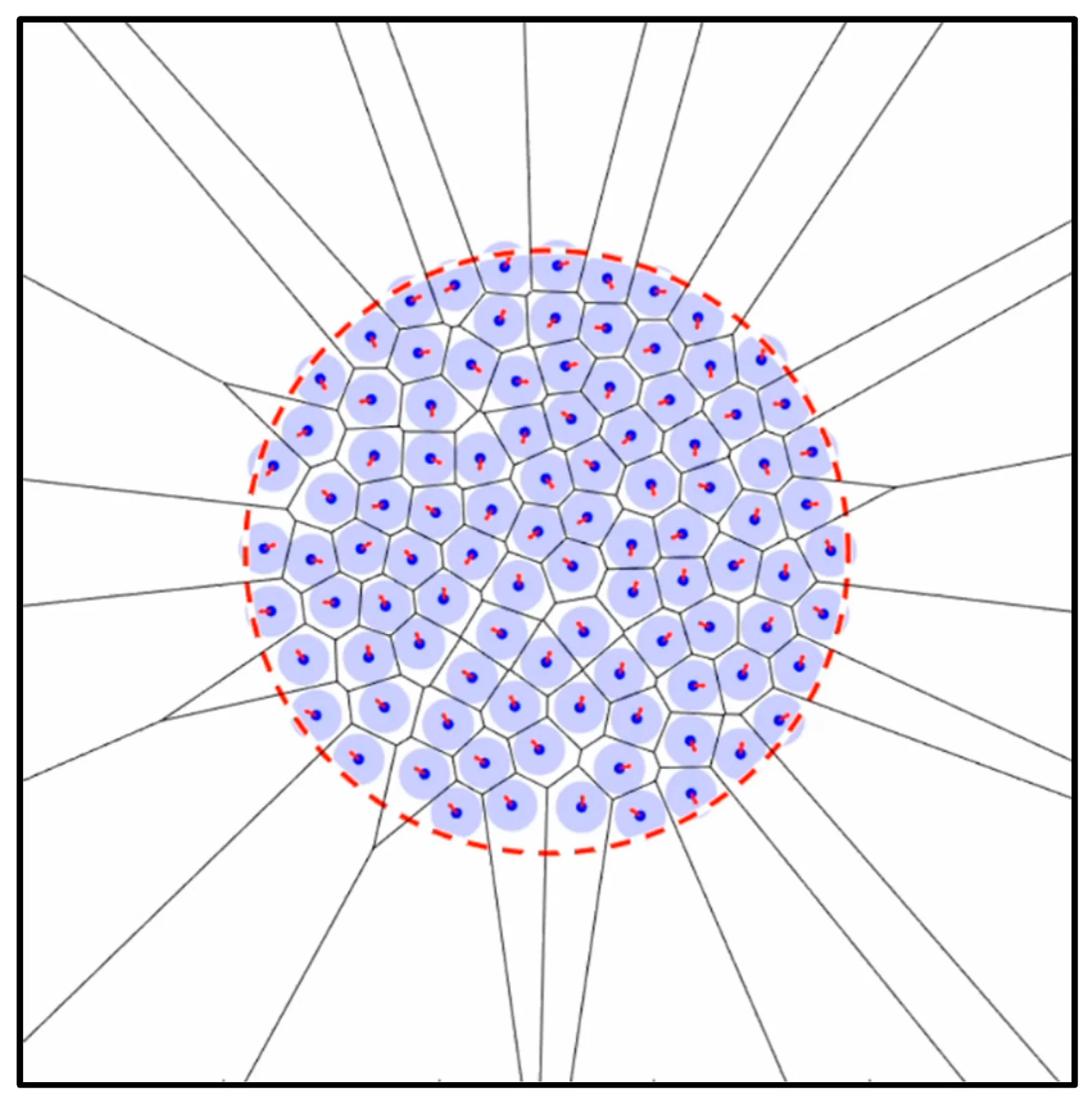
Voronoi tessellation of the particle packing configuration.

To compute local packing densities, we construct the Voronoi tessellation of the active particle configuration at a timestep (see Figure 4). Given a set of particle positions $\left\{x_{i}\right\}$, the Voronoi diagram divides the space into Voronoi cells, where each cell contains all points closer to a particular particle than to any other. However, some Voronoi cells extend infinitely due to the boundary effect, making density calculations impractical. To avoid this infinity issue, we consider only particles with fully enclosed Voronoi cells and the particles in the centre region of the final packing, excluding those whose cells extend beyond the confined region. Similar treatments have been applied in analysing the packing structures obtained by numerical simulations or experiments [46,47]. The local density is then obtained by taking the ratio of the area of the particle to the Voronoi cell area. The local density of the system is given by $\rho_{local}$ = $\;A_{i}/A_{Vor,i}$, where $A_{Vor,i}$ represents the area of the Voronoi cell associated with a given particle, and $A_{i}$ is the area of the particle, calculated as $\pi R_{r}^{2}$. The global packing density is the sum of the areas of particles fully enclosed by internal Voronoi cells, normalised by the total area of those cells. The global packing density (ρ) is defined as

(7) $$\rho =\frac{\sum_{i=1}^{N_{int}}A_{i}}{\sum_{i=1}^{N_{int}}A_{Vor,i}}=N_{int}\frac{A_{i}}{\sum_{i=1}^{N_{int}}A_{Vor,i}}=N_{int}\frac{1}{\sum_{i=1}^{N_{int}}\frac{1}{\rho_{loc,i}}},$$

where $N_{int}$ is the total number of fully enclosed internal Voronoi cells within the confined boundary $R_{b}$. Overall, employing Voronoi tessellation enables precise, particle-level measurement of density fluctuations and local structural heterogeneity, which are essential for understanding packing and jamming phenomena in the system.

## 3. Results and Discussion

### 3.1. Packing Density

The effects of the controlling variables on the packing of active particles are studied in terms of packing density, with the macroscopic parameter first.

#### 3.1.1. Effect of $R_{b}$ and $R_{r}$ with and Without Alignment

**Figure 5:**
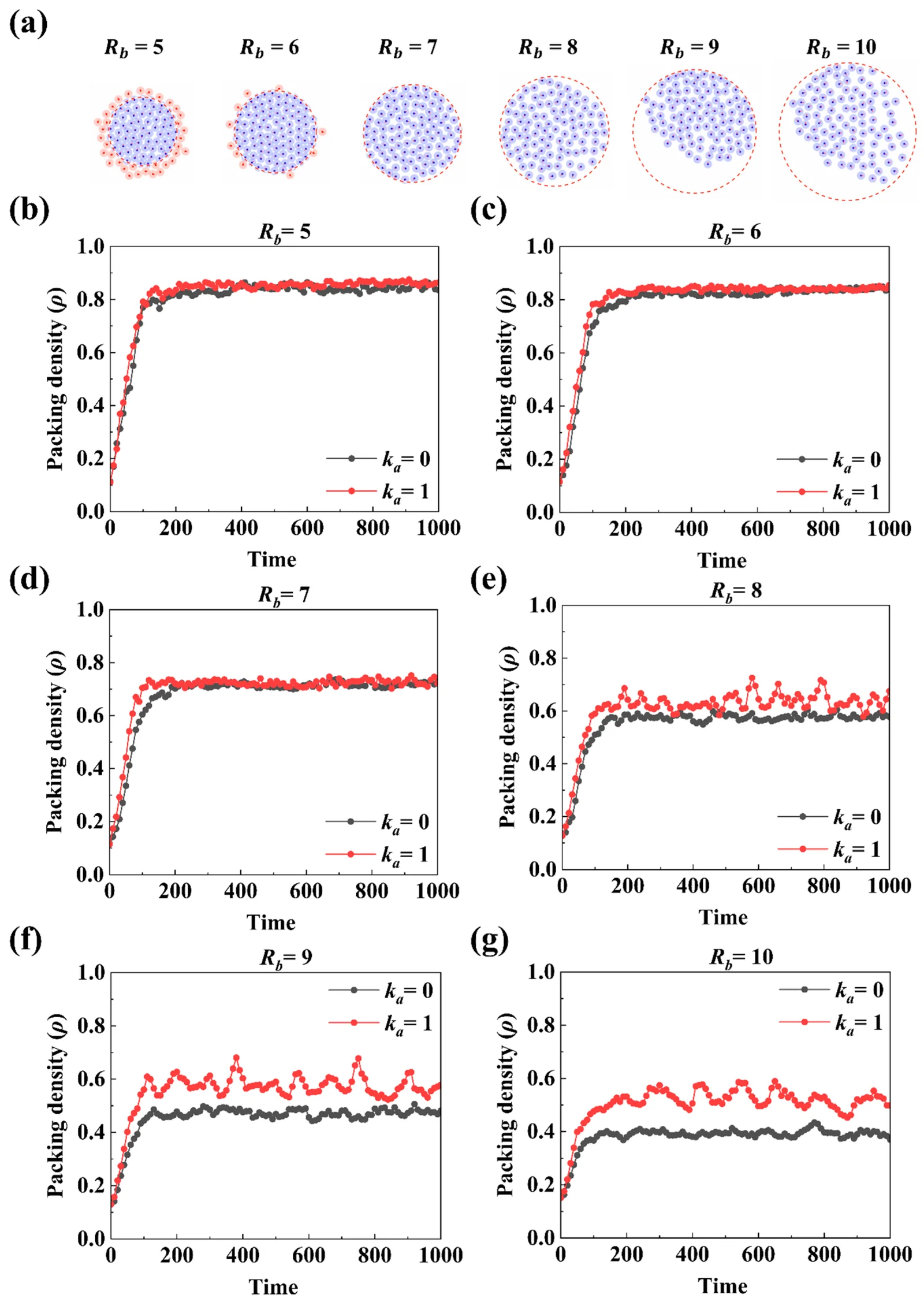
(**a**) Simulation snapshots at t = 1000 for different boundary radii with η = 0.5, $R_{r}$ = 1.2, and $k_{a}$ = 1. (**b**–**g**) Packing density as a function of time under different $R_{b}$ = 5, 6, 7, 8, 9, and 10, respectively, under $k_{a}$ = 0 and $k_{a}$ = 1.

The packing configurations at the final simulation timestep (*t* = 1000) under different boundary radii ($R_{b}$), a constant $R_{r}$ of 1.2, and η = 0.5 are shown in Figure 5a. For the smallest boundary radius ($R_{b}$ = 5), particles are packed densely inside the boundary, and a few particles are outside the confining region but cannot enter the region. When the boundary radius is increased to $R_{b}$ = 7, all particles can enter the boundary with a relatively dense packing structure with minimal voids. However, upon further increasing $R_{b}$ above 7, all particles not only enter the boundary but also have free space to move in the boundary. Although they still move together due to the local interactions, the voids between them are visually larger than those of $R_{b}$ < 7. These results show that with increasing boundary size, the packing of active particles may undergo a transition from crowded packing to loosely organised states.

In addition, the influence of alignment on packing density has been evaluated by setting $k_{a}$ = 0 and $k_{a}$ = 1. Figure 5b–g presents the temporal evolution of the packing density (ρ) under different boundary sizes with $k_{a}$ = 0 and $k_{a}$ = 1. In general, *ρ* increases over time as particles are driven toward the confining boundary, reaching a plateau at about t = 200 s. However, the values of ρ are different under different conditions. When $R_{b}$ < 7, ρ fluctuates very little and is almost the same under $k_{a}$ = 0 and $k_{a}$ = 1. In particular, when $R_{b}$ = 5, *ρ* reaches a stable value of 0.85, which is the closest to previous studies on self-propelled discs [48] where a jamming transition occurs when the packing density is about 0.88. The relatively lower packing density here is because of different interaction rules.

When $R_{b}$ increases to 7, the stabilised value of ρ decreases to about 0.75. The further increase of $R_{b}$ to 8 results in a more pronounced reduction in ρ and more significant differences between the two alignment conditions. Specifically, for $k_{a}$ = 0, ρ stabilises at a lower value near 0.58, while for $k_{a}$ = 1, ρ fluctuates between 0.58 and 0.7, with an average value of about 0.65. Similar enhancements of packing efficiency due to alignment have been reported in active matter and self-propelled rod systems, where collective ordering allows for denser configurations in moderately confined spaces [49]. Moreover, the effect of alignment is further enhanced with increasing $R_{b}$. As $R_{b}$ increases to 10, for $k_{a}$ = 1, ρ periodically changes between 0.55 and 0.6, but for $k_{a}$ = 0, ρ drops significantly to about 0.4 and is more stabilised. The increasing disparity between the case of $k_{a}$ = 0 and that of $k_{a}$ = 1 with larger $R_{b}$ underscores the enhanced role of alignment in less confined environments. Moreover, the active particles with alignment show a more obvious periodic change in packing density, which is similar to the cyclic expansion and shrinkage in some active systems [50].

**Figure 6:**
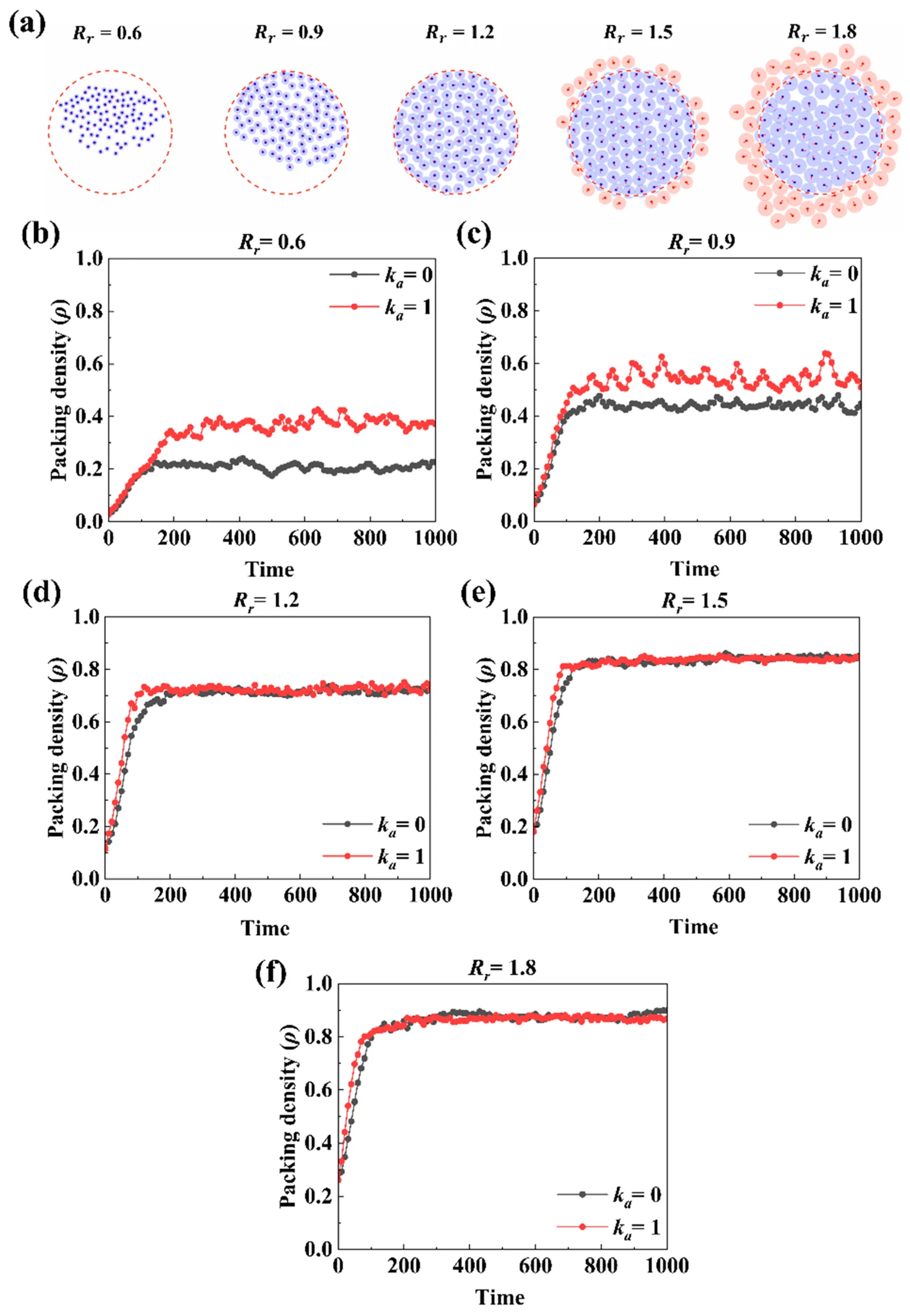
(**a**) Simulation snapshots at t = 1000 under different repulsion radii ($R_{r}$) with η = 0.5, $R_{b}$ = 7, R = 6 and $k_{a}$ = 1. (**b**–**f**) Packing density as a function of time under different $R_{r}$ = 0.6, 0.9, 1.2, 1.5, and 1.8 under $k_{a}$ = 0 and $k_{a}$ = 1.

Furthermore, with $R_{b}$ fixed at 7, the effect of $R_{r}$ on the packing behaviour is shown in Figure 6. It can be seen that the effect of increasing $R_{r}$ is opposite to that of increasing $R_{b}$. In particular, Figure 6a shows that at the final state, with increasing $R_{r}$, the system changes from a loosely organised packing to a densely jammed packing in the confined space with fixed $R_{b}$. Figure 6b–f shows that at a lower $R_{r}$, particle alignment facilitates higher ρ but when $R_{r}$ increases to 1.2, the alignment becomes less effective. In general, ρ increases with increasing $R_{r}$ and reaches a maximum of about 0.85, similar to that of varying $R_{b}$ with fixed $R_{r}$.

**Figure 7:**
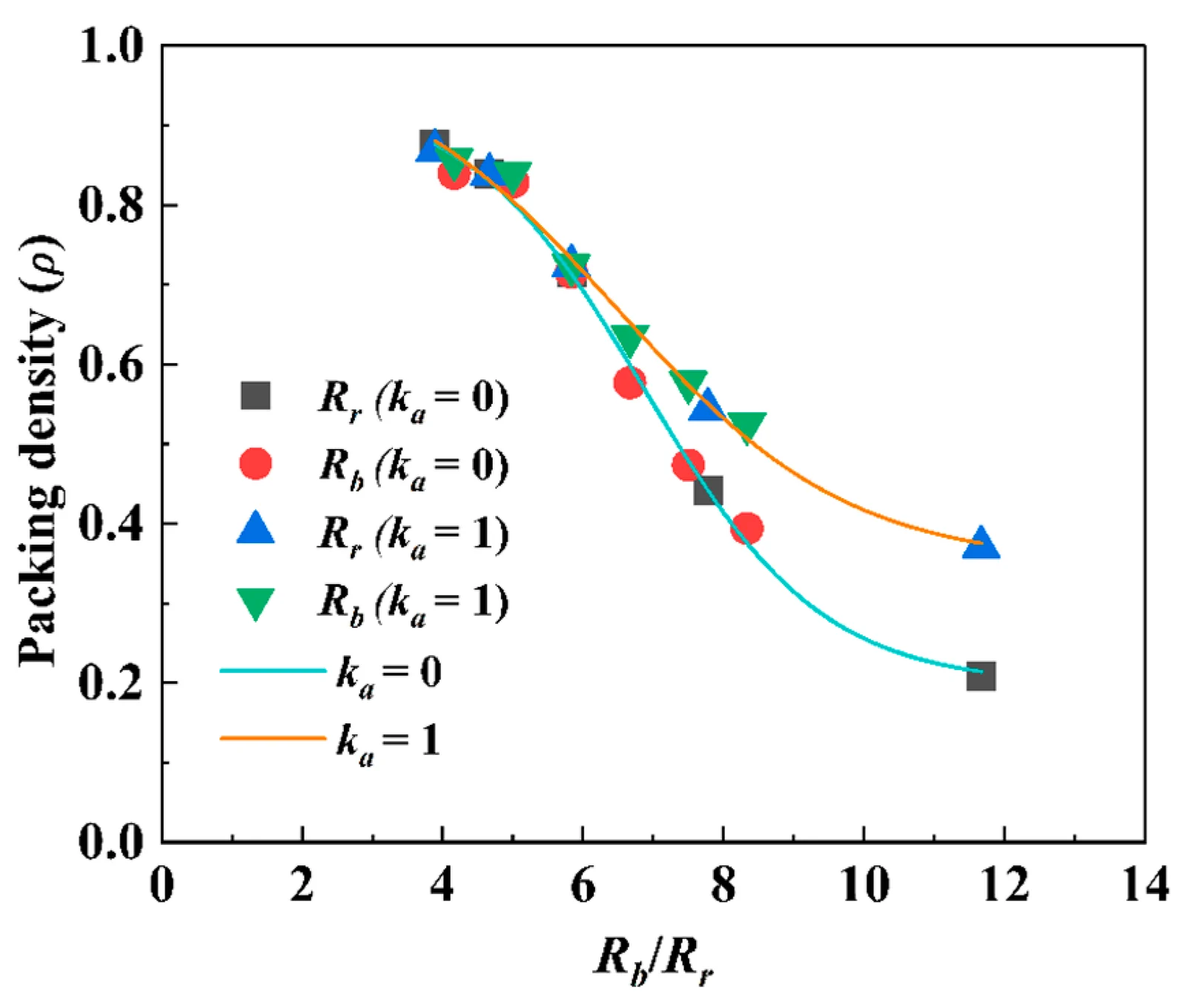
Packing density as a function of $R_{b}/R_{r}$ under $k_{a}$ = 0 and $k_{a}$ = 1 with η = 0.5. The lines connecting the data points are for visual aid only.

Figure 7 shows that the effects of $R_{b}$ and $R_{r}$ can be unified as that of the dimensionless ratio $R_{b}/R_{r}$. As this ratio increases, packing density decreases. When $R_{b}/R_{r}$ is small, the alignment interaction does not change packing density much. When $R_{b}/R_{r}$ is large, the alignment interaction enhances packing density. Here, a clear difference is observed between the aligned and non-aligned conditions: as $R_{b}/R_{r}$ increases, $k_{a}$ = 1 shows higher packing density compared to $k_{a}$ = 0. This suggests that velocity alignment can enable particles to occupy space more efficiently as the degree of confinement decreases. The present results are consistent with previous observations that confinement can strongly reshape the organisation of self-propelled and self-aligning particles [21]. In the present study, the confinement effect is introduced through the ratio between boundary radius and repulsion radius rather than trap steepness in [21]. Nevertheless, a comparable confinement-controlled transition is observed: when the effective packing space is small, the system is geometrically jammed and the alignment interaction has little influence on packing density; when the packing space is enlarged, the particles become less constrained, and alignment can increase the packing density. This suggests that boundary-mediated reorganisation is a common mechanism in confined active systems, while the present work further quantifies this effect using packing density rather than only polar or hexagonal order.

#### 3.1.2. Effect of Noise Strength on Packing Density

**Figure 8:**
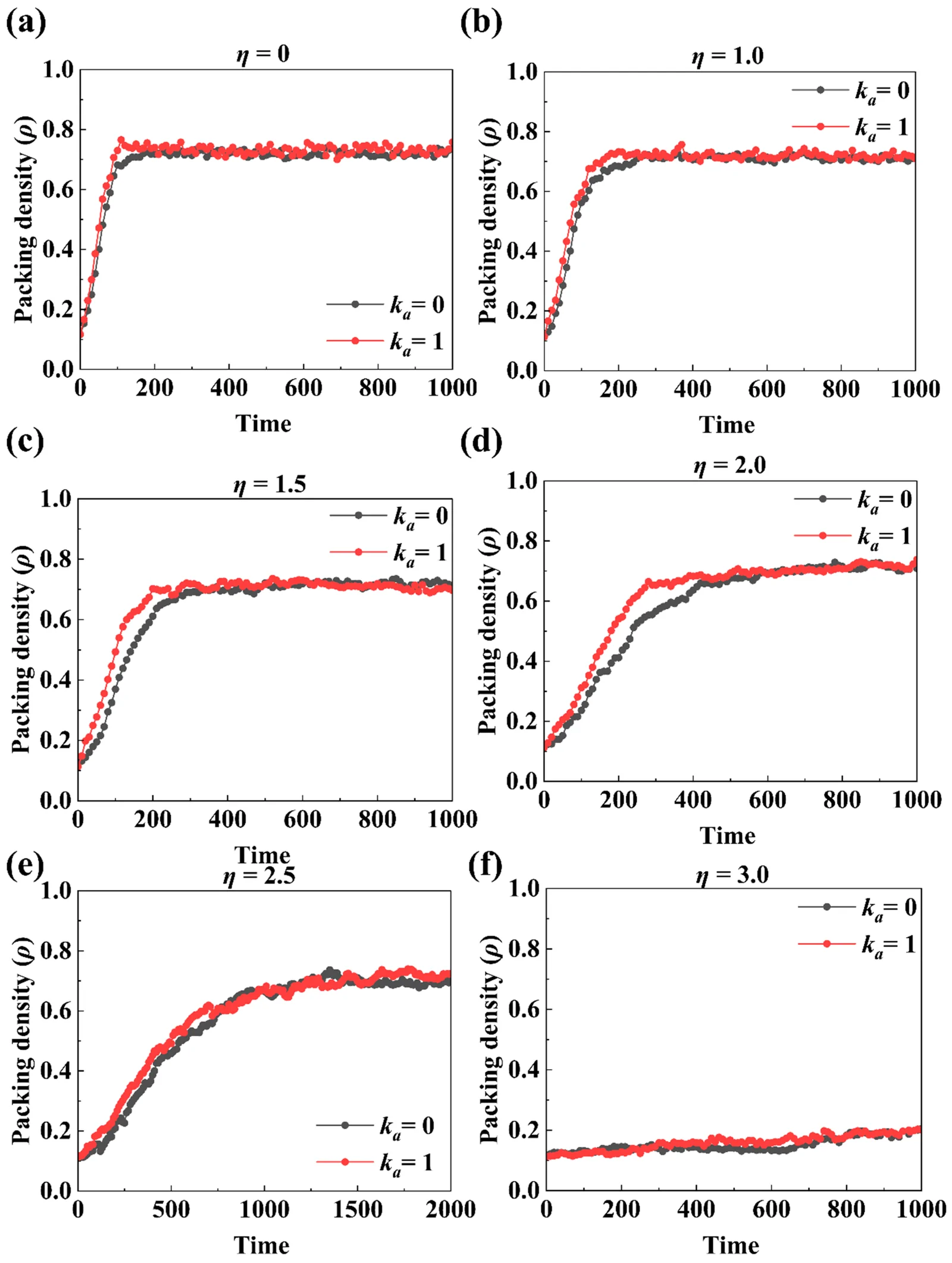
Packing density as a function of time under different noise strengths (η), with $k_{a}$ = 0 and $k_{a}$ = 1. Panels (**a**–**f**) correspond to increasing noise strengths from η = 0.0 to 3.0. The other parameters are fixed R = 6, $R_{r}$ = 1.2, and $R_{b}$ = 7.

For Vicsek systems, noise strength is an important controlling variable for collective behaviour. Here, we further investigate its effect on the packing of active particles in a confined boundary. The evolution of packing density (ρ) as a function of noise strength (*η*) over time under $k_{a}$ = 0 and $k_{a}$ = 1 with constant $R_{b}$ = 7 and $R_{r}$ = 1.2, is illustrated in Figure 8.

Similar to the previous observation, *ρ* generally increases rapidly at the beginning and reaches a steady state with slight fluctuations after a certain time. When *η* increases from 0 to 2.0, *ρ* at the final stage just decreases slightly, and the values at $k_{a}$ = 0 and $k_{a}$ = 1 are very close, suggesting neither *η* nor alignment is effective on the final packing density. However, increasing *η* prolongs the time to attain the steady state. Upon increasing *η* to 1.5 and 2.0, systems with alignment ($k_{a}$ = 1) attain steady-state density more rapidly than those without alignment ($k_{a}$ = 0), indicating the conducive effect of alignment on packing, likely due to the collective motion counteracting the dispersive effects of noise. As *η* increases to the critical threshold (*η* = 2.5), both systems require a longer duration to reach steady state ρ. Beyond this noise strength level, the system undergoes a sharp transition, and ρ plummets and stabilises at much lower values (between 0.15 and 0.2), irrespective of the alignment parameter. This sharp decrease indicates that excessive noise strength overwhelms the attraction toward the boundary, thereby disrupting the formation and maintenance of dense packing, consistent with observations of high dispersive behaviour in active particle systems [12].

These results highlight the critical role of both noise strength and alignment in determining the dynamics and steady-state structure of active matter when the boundary is confined ($R_{b}$ = 7). At low noise strength, packing is efficient, and alignment has a marginal effect. At moderate noise strength, alignment expedites the attainment of steady states. At high noise strength, particles cannot be effectively attracted into the boundary, so the packing density remains almost the initial value.

**Figure 9:**
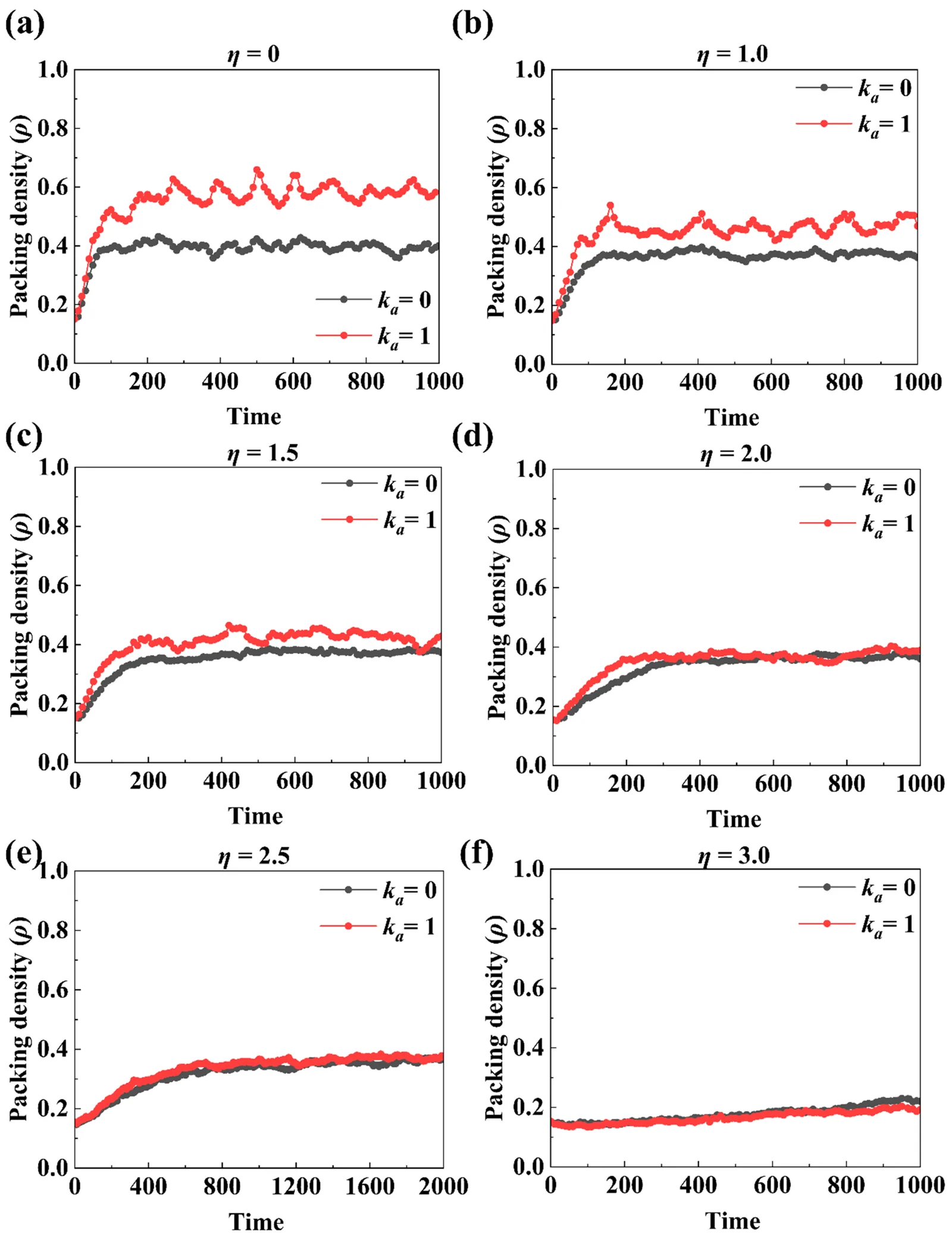
Packing density as a function of time under different noise strengths (η), with $k_{a}$ = 0 and $k_{a}$ = 1. Panels (**a**–**f**) correspond to increasing noise strengths from η = 0.0 to 3.0. The other parameters are fixed R = 6, $R_{r}$ = 1.2, and $R_{b}$ = 10.

As shown in Figure 8a–f, a small $R_{b}$ suppresses the effect of alignment on *ρ*; we therefore extended our analysis to a larger boundary radius ($R_{b}\;$ = 10). As shown in Figure 9a–f, ρ shows much higher sensitivity to noise strength than those for $R_{b}\;$= 7. At low noise strength (*η* = 1), the aligned system maintains a significantly higher packing density compared to the non-aligned case, demonstrating the enhanced structural organisation facilitated by the alignment interaction. As the noise strength level increases (*η* = 2.5), the overall packing density decreases monotonically, and the disparity between aligned and non-aligned systems is progressively reduced. Beyond a threshold noise amplitude (*η* = 3), the packing density reaches its lowest value, which is also due to the boundary attraction becoming ineffective due to the large noise strength.

#### 3.1.3. Phase Diagram of ρ as a Function of $R_{b}$/$R_{r}$ and η

**Figure 10:**
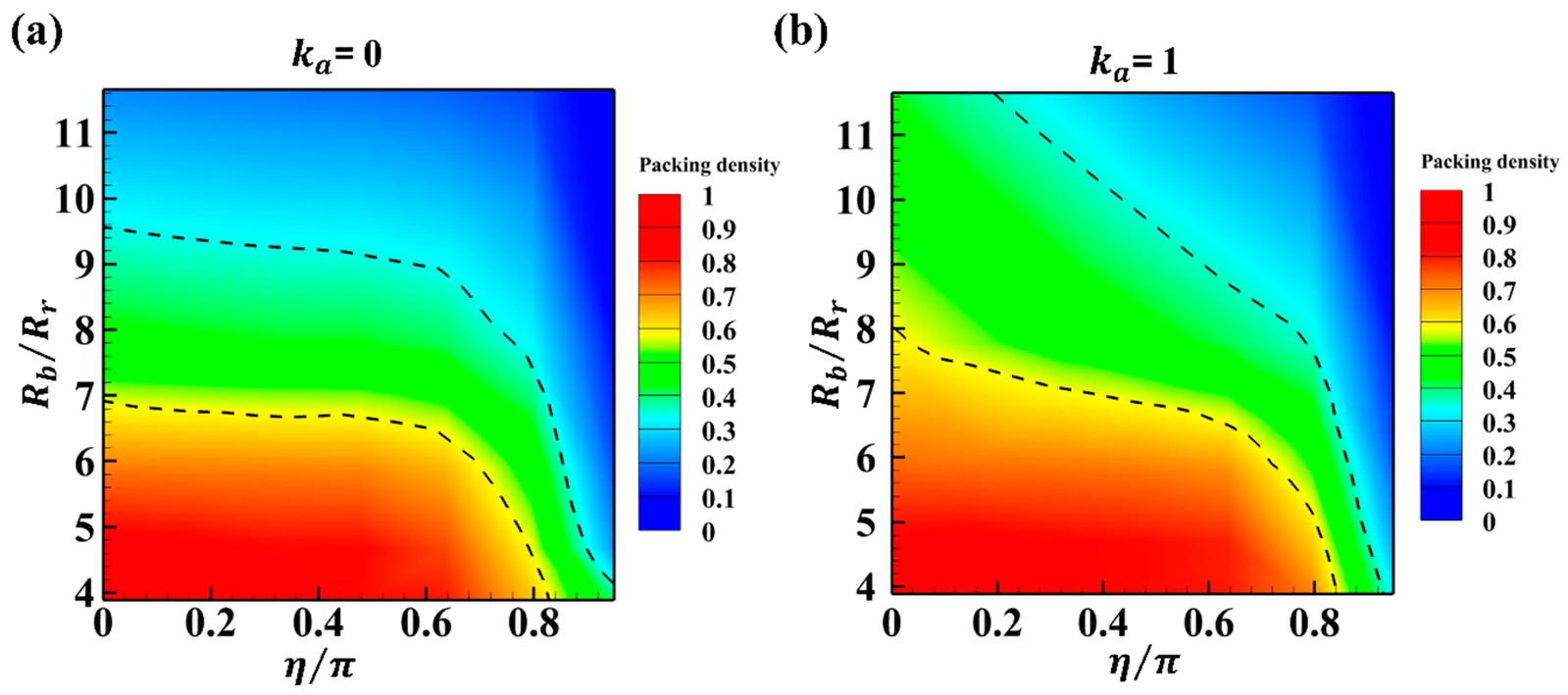
Packing density as a function of normalised boundary size and noise strength. (**a**). $k_{a}$ = 0. (**b**). $k_{a}$ = 1.

Figure 10 demonstrates the contour plot of packing density (ρ) as a function of normalised boundary radius ($R_{b}/R_{r}$) and normalised noise strength (η/π), with a comparison of both the non-aligning ($k_{a}$ = 0) and aligning ($k_{a}$ = 1) scenarios. For both scenarios, three distinct regimes of ρ are evident in the contour plot. The first regime, observed at low values of both $R_{b}/R_{r}$ and η/π, depicted as the darkest red region, corresponds to the highest ρ. In this region, both noise strength below a threshold and alignment have limited effects on *ρ*, indicating particles are jammed by the strong confinement. It is worth noting that very high noise strength can break this jammed region.

The middle region, which is mainly coloured green, corresponds to an increase of $R_{b}/R_{r}$ from the jammed region. With a spatial boundary, ρ decreases. In this region, noise strength shows little effect on ρ when it is not close to the maximum value. However, the alignment interaction obviously expands this region into the left upper corner, indicating that alignment increases packing density when the boundary is spacious enough ($R_{b}/R_{r}$ > 10), but such an increase is only effective when the noise strength is relatively small. This shows that the alignment interaction, as a local order mechanism, can also affect the packing density, but the noise strength needs to be sufficiently small, which is similar to the fact that local alignment leads to order transitions with decreasing noise strength in the Vicsek system [12]. For $k_{a}$ = 0, a pronounced boundary is visible, characterised by a flat yellow contour line separating regions of intermediate and low ρ. This abrupt transition demonstrates the system’s sensitivity to confinement in the absence of collective alignment. In contrast, for the aligning case ($k_{a}$ = 1), the transition from high to intermediate ρ is more gradual. The dark red region fades smoothly as $R_{b}/R_{r}$ increases, indicating that alignment interactions partially mitigate the dilution effect caused by weaker confinement, thereby sustaining higher packing densities across a broader range of $R_{b}/R_{r}$.

Finally, the third regime, coloured blue, emerges at high noise strength levels η/π, where ρ drops to its lowest values. This region spans a wide range of $R_{b}/R_{r}$, demonstrating that excessive noise strength disrupts collective motion, leading to inefficient packing, regardless of the presence of alignment interactions. The dynamic phase diagrams are often reported for confined active matter [24,25]. The present phase diagram focuses on a structural view rather than dynamic flow states. The three regimes identified here—jammed dense packing, alignment-enhanced packing, and noise-strength-dispersed loose packing—therefore provide a complementary structural view of confined active matter. In particular, the results show that alignment can improve packing only in a partially relaxed confinement regime; under very strong confinement, the system is already geometrically jammed, whereas under very strong noise, collective organisation is destroyed.

### 3.2. Local Structure Analysis

#### 3.2.1. Local Density Distribution

**Figure 11:**
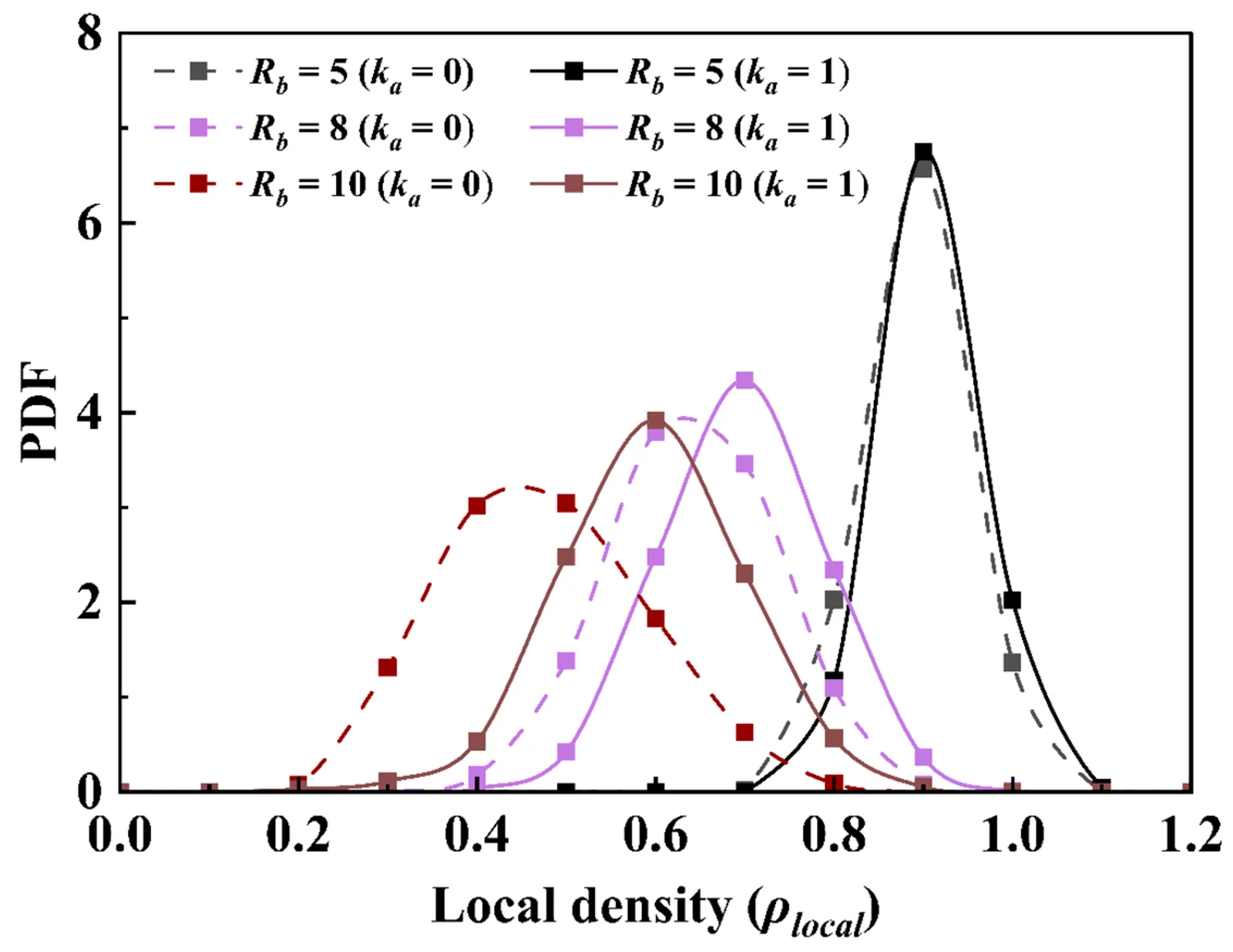
Distribution of local density under different $R_{b}$ and $k_{a}$ with η = 0.5, $R_{r}$ = 1.2, R = 6. The lines connecting the data points are for visual aid only.

The probability density function (PDF) of the local density ($\rho_{local}$) provides a quantitative measure of local structures. The shape and position of the PDF reflect the prevalence and magnitude of dense or sparse regions, as well as the uniformity or variability in particle distribution. Figure 11 shows the PDF of $\rho_{local}$ under different $R_{b}$ and $R_{r}$, and with or without alignment, which are based on the simulated snapshots from *t* = 200 to 1000.

When $R_{b}$ is small (5 to 7), the PDF is narrow and sharply peaked at high $\rho_{local}$, indicating the prevalence of dense local environments, whilst the presence or absence of alignment interactions has minimal effects on both the overall packing density and the microscopical packing structure. When $R_{b}$ increases, the distribution becomes broader and its peak value decreases, showing more diverse local structures. The distribution’s mode also shifts leftward, signalling a general decrease in local density as particles are less likely to become sterically trapped. In this $R_{b}$ range, the effect of alignment becomes obvious, making the distribution exhibit a higher peak and a rightward shift, showing that alignment generally improves local density. Across different cases, most distributions are approximately Gaussian, exhibiting a single peak and an approximately symmetric shape. This behaviour is consistent with normal-like distributions also reported in other active matter and particulate systems [17,46]. However, for $R_{b}$ = 5, the aligned case exhibits a small positive skewness. This is mainly because under this strongest confinement examined, the local-density distribution is narrow and concentrated close to the upper geometrical packing limit. These less frequent near-saturation configurations produce a short high-density tail and hence weak positive skewness. The small skewness and low heterogeneity indicate a ceiling effect within an otherwise uniformly dense packing, rather than the emergence of a strongly heterogeneous state.

**Figure 12:**
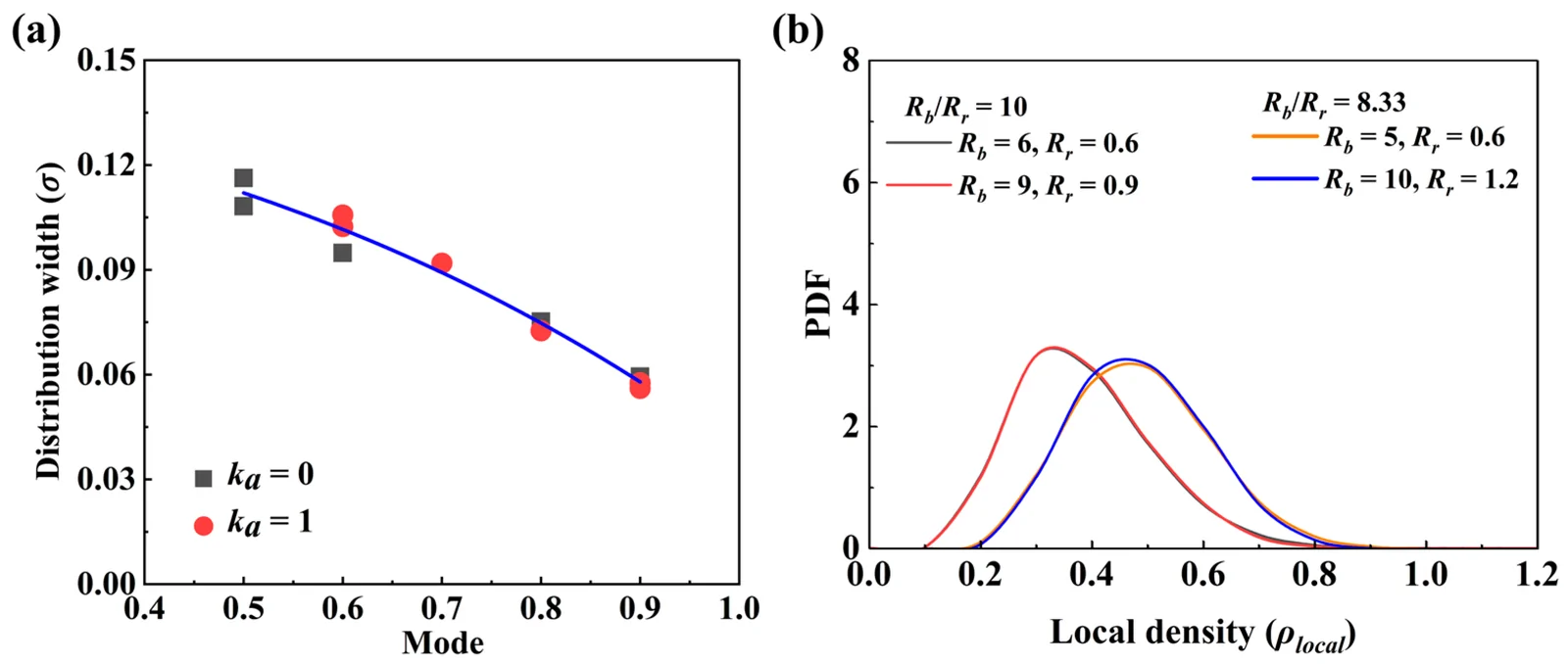
General correlation and self-similarity in local-density distributions. (**a**) Distribution width versus mode value for different alignment conditions $k_{a}$ = 0 and $k_{a}$ = 1. The lines connecting the data points are for visual aid only. (**b**) Comparison of local density distributions for different combinations of boundary radius ($R_{b}$) and repulsion radius ($R_{r}$) at fixed $R_{b}/R_{r}$.

We quantitatively compare the local density ($\rho_{local}$) mode value and distribution width as a function of $R_{b}$ under both conditions $k_{a}$ = 0 and $k_{a}$ = 1. The mode is determined by identifying the peak of the local density distribution, while the distribution width is measured by calculating either the standard deviation or the full width at half maximum. These data sets always follow a normal distribution $\frac{1}{\sigma \sqrt{2\pi }}\;e^{\left(-\frac{{\left(x-\mu \right)}^{2}}{{2\sigma }^{2}}\right)}$, where μ represents the mode and σ describes the distribution width. As shown in Figure 12a, for both $k_{a}$ = 0 and $k_{a}$ = 1, the distribution width decreases with increasing mode value, demonstrating a uniform trend that the local structure becomes more uniform at higher packing density. To examine whether the local-density distributions exhibit self-similarity, we compared distributions for different combinations of boundary radius ($R_{b}$) and repulsion radius ($R_{r}$) at fixed $R_{b}/R_{r}$ (see Figure 12b). The distributions show close overlap for the tested cases, indicating approximate self-similarity.

**Figure 13:**
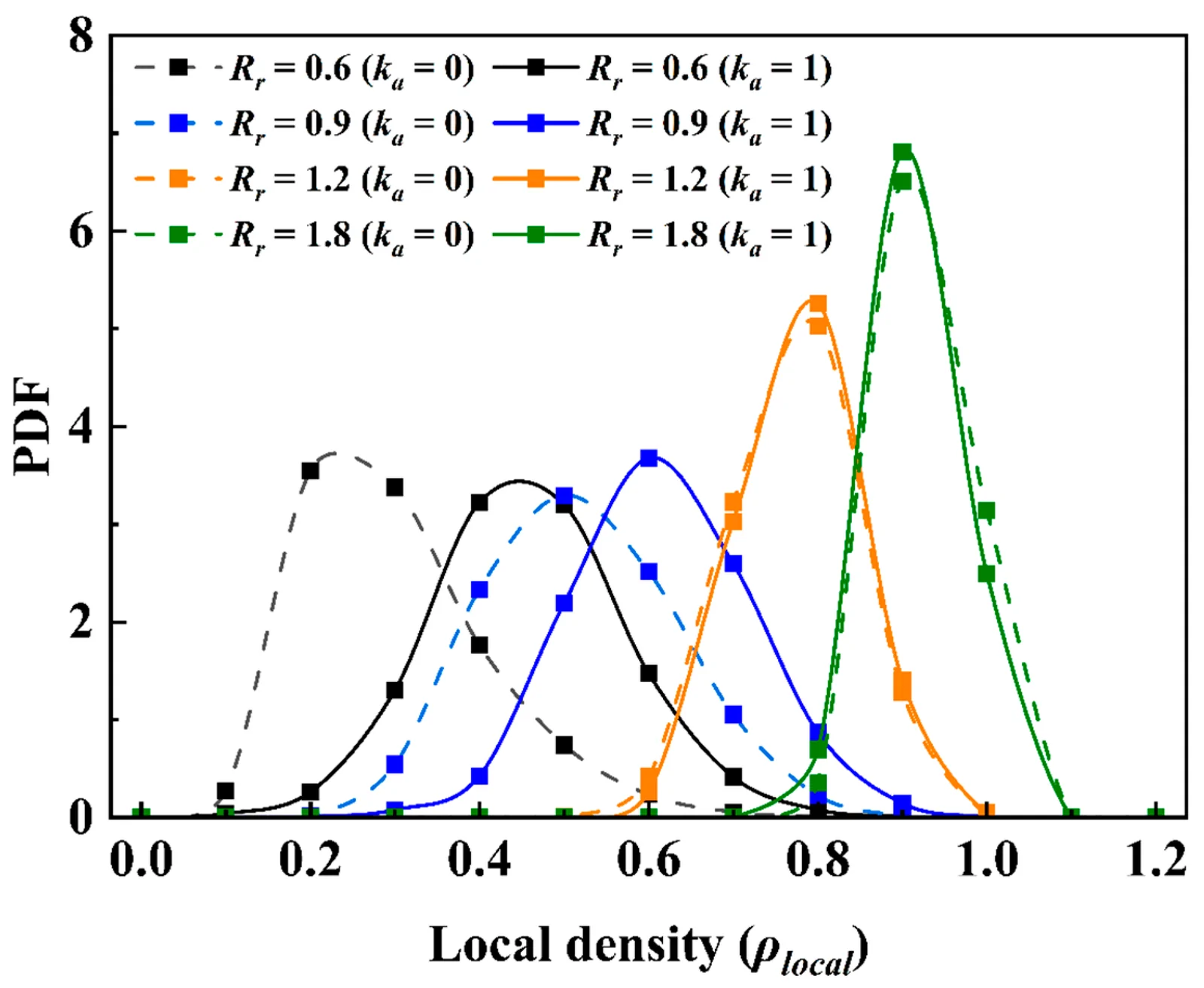
Distribution of local density under different $R_{r}$ with constant η = 0.5, $R_{b}$ = 7, R = 6 under $k_{a}$ = 0 and $k_{a}$ = 1.

Figure 13 illustrates the $\rho_{local}$ distribution under different $R_{r}.$ Most of the distributions are unimodal and approximately bell-shaped, with a single peak. The exception is the non-aligned case at $R_{r}$ = 0.6, which exhibits pronounced positive skewness and an extended tail toward higher local densities and is better described by the log-normal model. This behaviour is qualitatively related to motility-induced local clustering [18]: persistent propulsion and repeated steric encounters occasionally generate compact particle configurations within an otherwise dilute packing. However, the distribution remains unimodal, and no macroscopic dense–dilute coexistence is identified; therefore, the result is interpreted as MIPS-like intermittent local clustering rather than fully developed MIPS. At fixed $R_{b}$ = 7, this case $R_{r}$ = 0.6 corresponds to the largest $R_{b}$/$R_{r}$, representing the weakest effective confinement examined. As $R_{r}$ increases, the excluded region becomes larger and local spacing is regulated more uniformly, suppressing the high-density tail and restoring approximately Gaussian statistics.

Increasing $R_{r}$ demonstrates a similar effect as decreasing $R_{b}$. At higher $R_{r}$ (>1.0), the distribution becomes narrower and more sharply peaked at higher densities with increasing $R_{r}$, and the alignment interaction does not have significant effects. At lower $R_{r}$ (0.6 and 0.9), the distribution peak shifts leftwards, with decreasing peak height and increasing peak width, indicating that the local structures are more diverse. Moreover, when $R_{r}$ is low (0.6 and 0.9), the alignment interaction does not affect the peak height and width much, but makes the peak locate at a higher value of $\rho_{local}$. This also shows that the alignment interaction generally improves $\rho_{local}$ when it is more widely distributed. These observations are consistent with the previous study, which demonstrates the critical influence of confinement geometry, where active particles in larger or less restrictive domains exhibit less pronounced local density due to decreased crowding [51,52].

**Figure 14:**
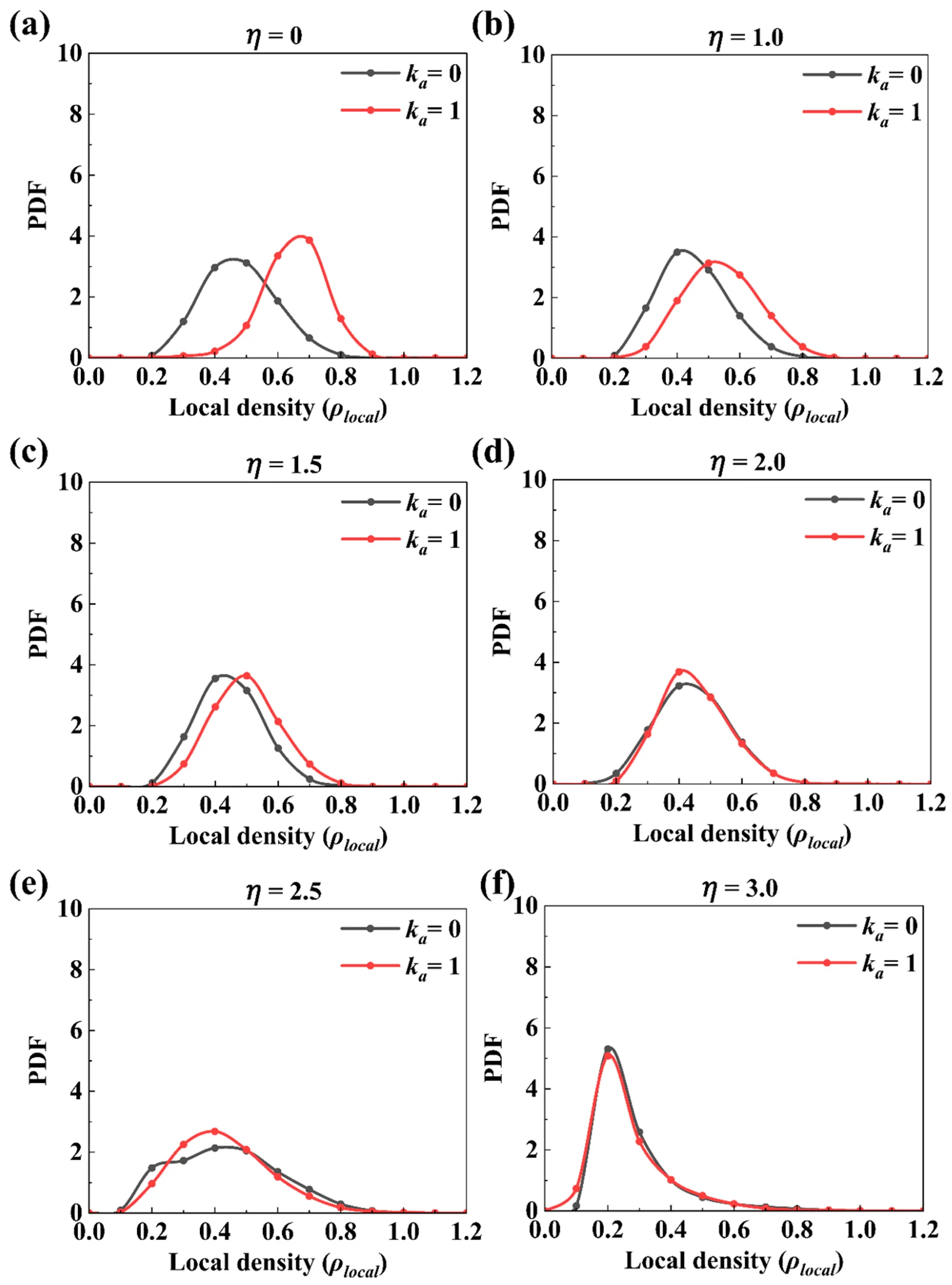
PDF of the local density ($\rho_{local}$) distribution for different η with $k_{a}$ = 0 and $k_{a}$ = 1. The distributions are calculated over the time interval 200 to 1000. Panels (**a**–**f**) correspond to increasing noise strengths from η = 0.0 to 3.0. The other parameters are fixed at $R_{r}$ = 1.2, $R_{b}$ = 10, and R = 6.

In active matter, the alignment will be weakened by increasing noise strength. Here, the $\rho_{local}$ distributions under different noise strengths and $k_{a}$ are illustrated in Figure 14a–f, which are also calculated based on the snapshots from *t* = 200 to *t* = 1000. At low noise strength, the alignment ($k_{a}$ = 1) markedly narrows the $\rho_{local}$ distributions and increases their peak heights compared to $k_{a}$ = 0, reflecting stable and dense local structures sustained by persistent directional coherence.

However, as *η* increases, stochastic reorientations compete with alignment, intermittently breaking dense structures. This leads to broadening and a leftward shift in the $\rho_{local}$ distributions, with reduced peak heights in the aligned systems. For $k_{a}$ = 0, the distribution develops a low-density shoulder at the sampled value η = 2.5. This is interpreted as a crossover regime in which stochastic reorientation has disrupted some local packed structures while others remain comparatively dense. The partially disrupted configurations form a minority low-density population and generate the shoulder. At η = 3.0, disruption becomes widespread, and the low-density population becomes dominant. Consequently, the two contributions are no longer resolved as a distinct shoulder; instead, the remaining denser configurations form the high-density tail of a broad positively skewed distribution. The continuous increase in the heterogeneity measure from η = 2.0 to 3.0 supports a high-noise crossover rather than a distinct structural phase occurring only at η = 2.5. At sufficiently high *η*, alignment effects vanish, and the $\rho_{local}$ distributions of aligned and non-aligned systems converge. Quantitative fitting also indicates that, for most low and intermediate noise strengths, the distributions are approximately Gaussian, whereas, with high noise strength (η = 3), the distributions are positively skewed and described by a log-normal distribution, suggesting enhanced heterogeneity in the local-density field (see Supplementary Materials). This suggests that increased *η* disrupts the formation of densely packed local structures, leading to a more heterogeneous distribution of particles.

#### 3.2.2. Coordination Number Analysis

Coordination number (CN) refers to the number of nearest neighbouring contacts per particle. It is an important parameter for describing the structural properties of particle–particle connections in packing [28,53]. As a metric, it is particularly useful for evaluating the degree of connectivity within a packing and directly influences macroscopic properties such as tensile strength [54,55], thermal conductivity [56], solid–solid reactions [57], phase formation [28] and permeability [58].

**Figure 15:**
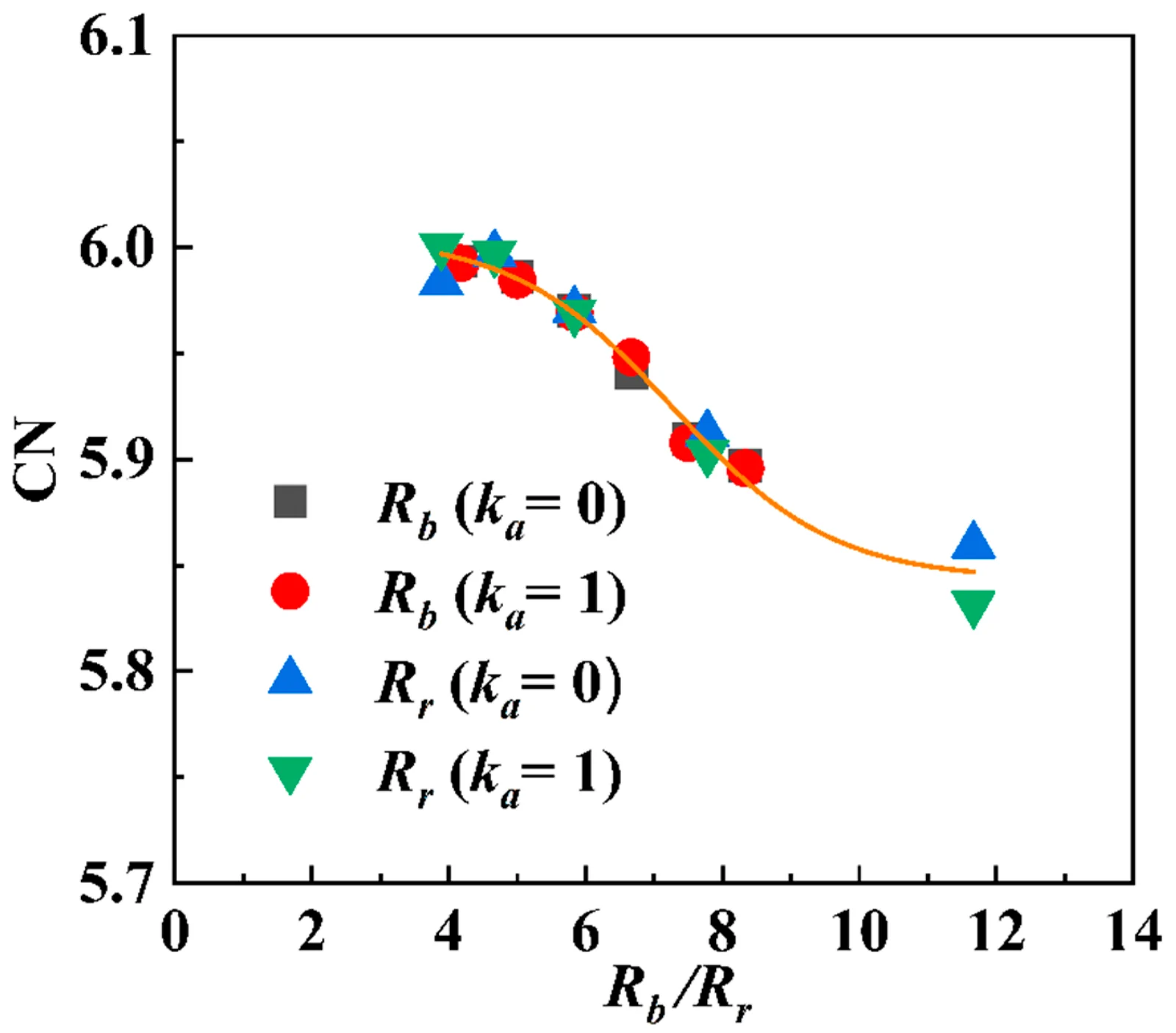
The average CN as a function of $R_{b}$/$R_{r}$. The lines connecting the data points are for visual aid only.

In active matter, CN is dynamic and fluctuates due to the constant movement of particles. Several approaches have been proposed for defining “neighbours,” typically relying on either a fixed metric distance [59] or topological criteria, such as those based on the construction of Voronoi tessellations [60]. In this study, we determine the coordination number for each particle by identifying the number of contiguous Voronoi cells sharing a face with the Voronoi polyhedron of the central particle. Figure 15 shows the average coordination number (CN) as a function of $R_{b}/R_{r}$. With increasing ratio $R_{b}/R_{r}$, CN decreases monotonically, which is consistent with the variation of overall packing density and results from the looseness of the confinement. Interestingly, the maximum average CN is about 6, which is in accordance with the hexagonal packing of 2D discs [61]. This further confirms that when the confinement is strong, the structure of the system is dominated by the geometrical effect, but the active effect (alignment interaction) is minimal.

#### 3.2.3. CN Versus Global Packing Density

**Figure 16:**
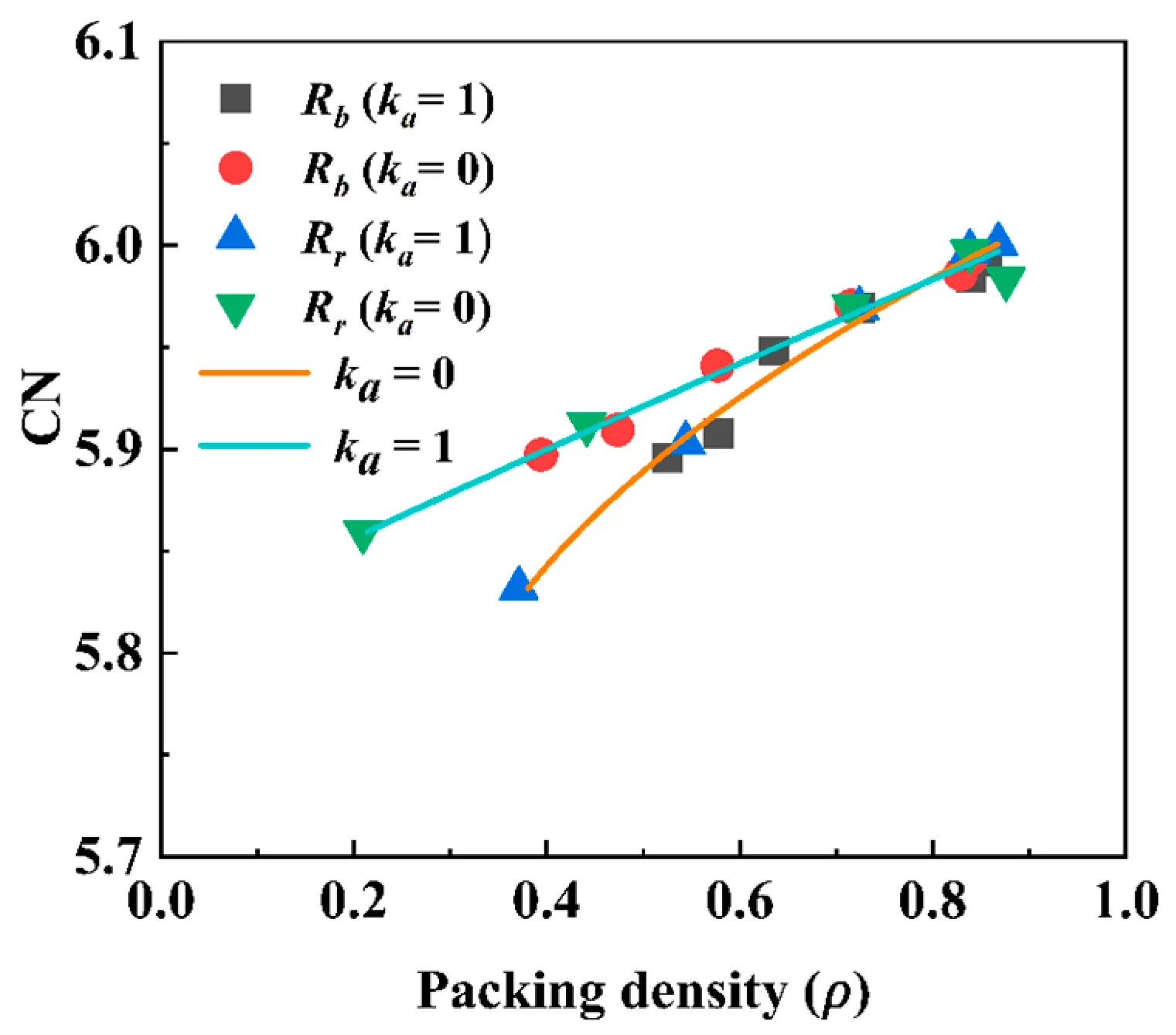
Combined effect of $R_{b}$ and $R_{r}$ on the relationship between packing density (ρ) and average CN (over *t* = 200 to *t* = 1000).

Figure 16 presents CN versus packing density (ρ) with varying $R_{b}$ and $R_{r}$ under both aligned and non-aligned conditions. Generally, CN increases with increasing ρ, similar to granular packings [61]. In addition, different data points collapse onto two lines representing $k_{a}$ = 0 and $k_{a}$ = 1, respectively, indicating a quasi-universality between macroscopic packing density and microscopic structures, as also shown in granular packings [46]. On the other hand, in the low packing density region under the same CN, ρ is higher for $k_{a}$ = 1 than for $k_{a}$ = 0, indicating that with the same number of neighbouring particles, the packing is more efficient with the local alignment. This should be because a particle will move more coherently with neighbouring particles and hence require less space, which is the micro-mechanism for the enhancement of particle density by alignment. Moreover, this also explains why the alignment increases packing density in Figure 7 although it does not affect the CN in Figure 15.

### 3.3. Polar Order Parameter

Since alignment similar to the Vicsek system is considered, the polar order φ is also an important property of the system. In the Vicsek model [12], the alignment interaction will lead to an order transition with increasing density and decreasing noise strength, and φ approaches approximately 1.0. Here, we investigate whether such an order transition also occurs in the studied packing system.

#### 3.3.1. Effect of Noise Strength on Order Parameter

**Figure 17:**
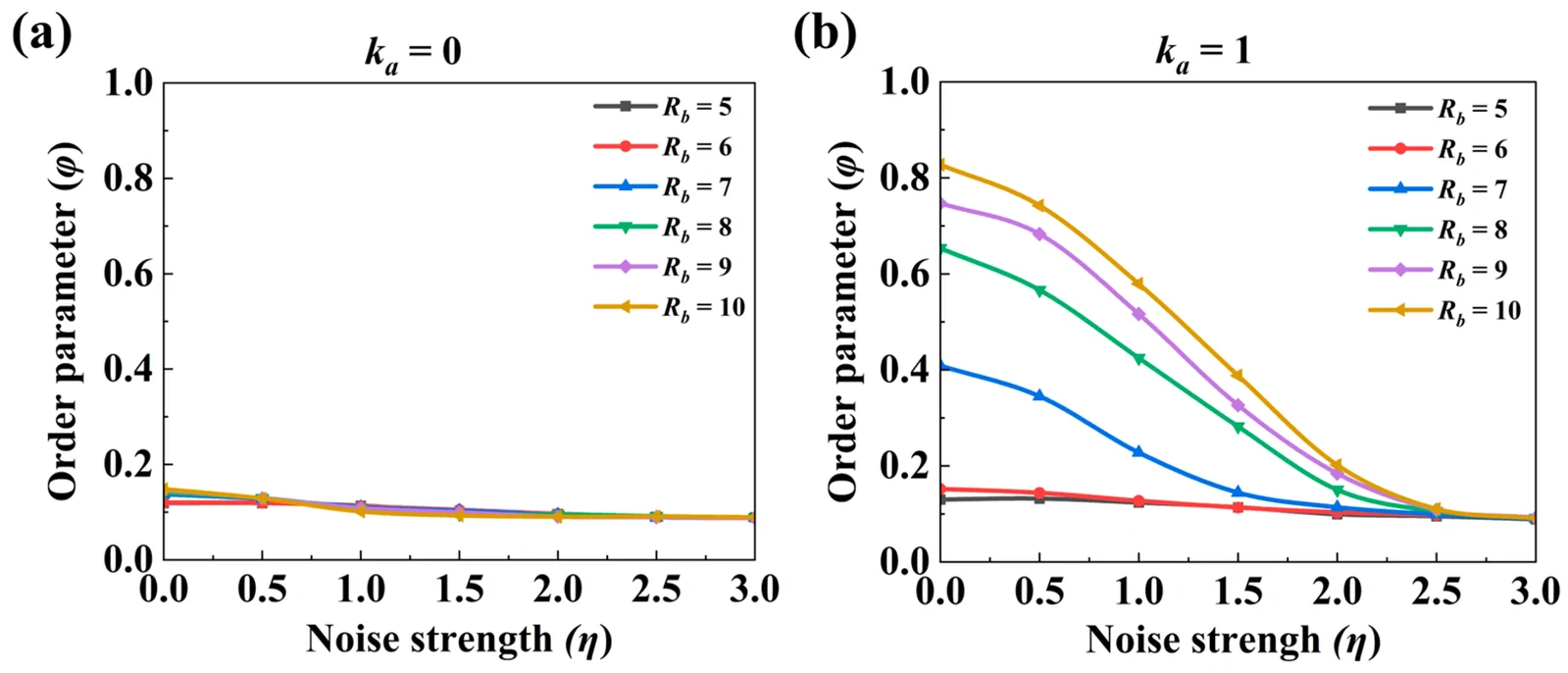
Effect of noise strength (η) on the order parameter (φ) for different boundary radii ($R_{b}$) with constant $R_{r}$ = 1.2. (**a**). $k_{a}$ = 0. (**b**). $k_{a}$ = 1.

We employ time-averaged data after the system has reached a statistically steady state. Figure 17 illustrates how φ varies under η without alignment ($k_{a}$ = 0) and with alignment ($k_{a}$ = 1). For $k_{a}$ = 0, the order parameter remains low regardless of $R_{b}$ and η, showing the system remains disordered in the velocity directions of particles. In contrast, when alignment interactions are present ($k_{a}$ = 1), φ increases with decreasing η when $R_{b}$ is greater than 6, showing a similar order transition as that in the Vicsek system [12,62]. On the other hand, confinement can regulate this polar-order phase transition. In particular, under the same η, φ is smaller when $R_{b}$ is smaller. Notably, a critical point emerges around η = 2.5, beyond which the order parameter drops to the minimum for all $R_{b}$. This critical point indicates that for the combined effect of noise strength and confinement, η is still controlling the order transition of the system, whereas stronger confinement (smaller $R_{b}$) decreases φ generally.

#### 3.3.2. Effect of $R_{b}$ and $R_{r}$

**Figure 18:**
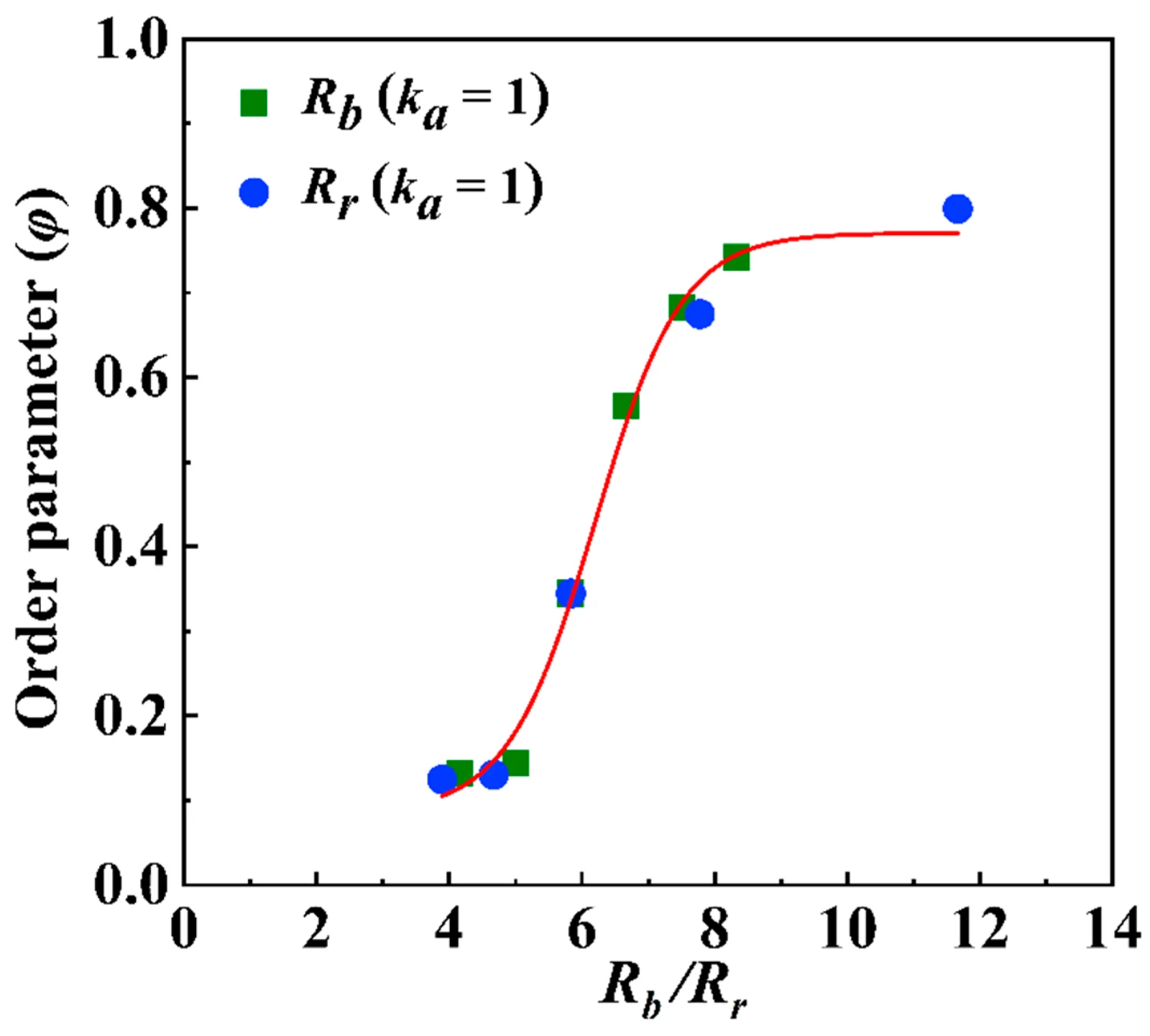
Effect of repulsion radius ($R_{r}$), and boundary radius ($R_{b}$) on the order parameter (φ) under $k_{a}$ = 1 and η = 0.5. The line connecting the data points is for visual aid only.

In order to separate the effect of confinement, we study φ as a function of $R_{b}/R_{r}$ with fixed η. As shown in Figure 18, η = 0.5, φ shows a uniform correlation with $R_{b}/R_{r}$ regarding the changes in $R_{b}$ and $R_{r}$. Interestingly, an order transition with $R_{b}/R_{r}$ can be clearly identified. In particular, φ remains low when $R_{b}/R_{r}$ is small, but it rises sharply after $R_{b}/R_{r}>$ 5 and saturates at about 0.8 when $R_{b}/R_{r}\geq$ 8. It quantitatively shows that the strong confinement not only jams the packing structure but also jams the polar order of the active particles when $R_{b}/R_{r}$ is below a threshold, and the polar order transition can also be incurred by varying $R_{b}/R_{r}$. The maximum φ (0.8) has a certain gap with that of the Vicsek system (above 0.9), which is probably because of the boundary and the repulsion force. Here, we observe a similar trend in the order parameter as seen in the Vicsek model when increasing the density [12].

#### 3.3.3. Packing Density vs. Order Number

**Figure 19:**
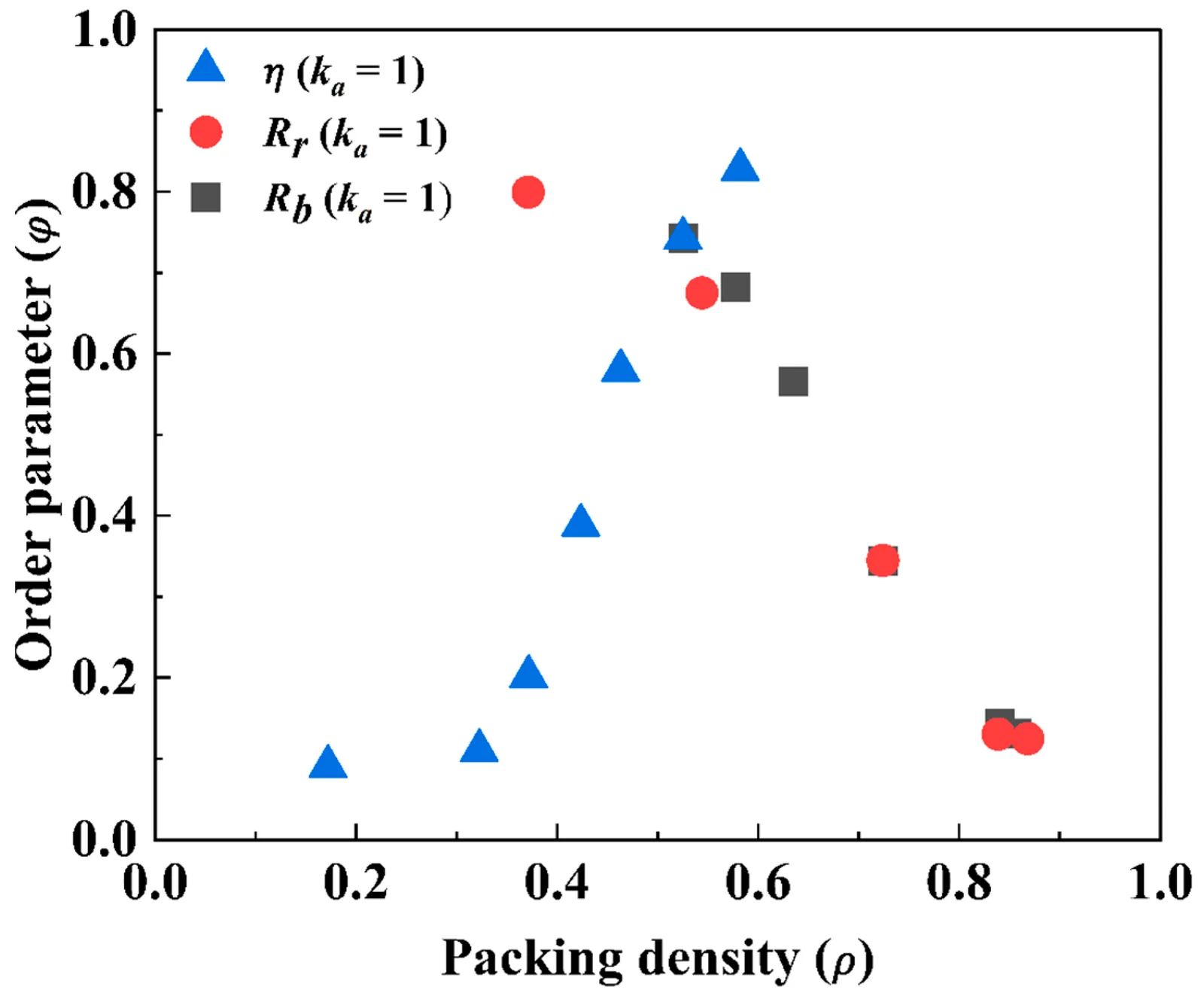
Order parameter (φ) versus packing density (ρ) for three parameter sweeps at $k_{a}$ = 1. η series (blue triangles): noise strength varies from 0 to 3 under $R_{b}$ = 7 and $R_{r}$ = 1.2. $R_{b}$ series (grey squares): boundary radius varies from 5 to 10 under η = 0.5 And $R_{r}$ = 1.2. $R_{r}$ series (red circles): repulsion radius varies from 0.6 to 1.8 under η = 0.5 And $R_{b}$ = 7.

To evaluate the combined effect of boundary radius, repulsion radius, and noise strength on packing density and the order parameter, we compared system behaviour across a range of parameter values. Figure 19 shows the relationship between packing density (ρ) and order parameter (φ) with varying system parameters. There are clearly two stages. First, we observe that the order parameter increases as the packing density increases, which results from decreasing noise strength at a high $R_{b}/R_{r}$. This suggests that when the boundary is spacious, packing density and order number are increased simultaneously. At this stage, φ reaches its highest value, close to 0.9, while ρ just reaches a medium value at about 0.6. Second, we find that further increasing ρ requires using the boundary effect, as ρ increases beyond 0.6 due to the decrease in $R_{b}/R_{r}$. At this stage, an increase in ρ is accompanied by a decrease in φ, suggesting a similar trend to the packing of granular particles where random close packing is achieved when the structure order parameter reaches its lowest value [63]. It is interesting to see that the polar order number shares the same trend as the structure order number, which indicates that there may be a universal relationship between the order and the packing density across passive and active particle systems.

#### 3.3.4. Phase Diagram of φ

**Figure 20:**
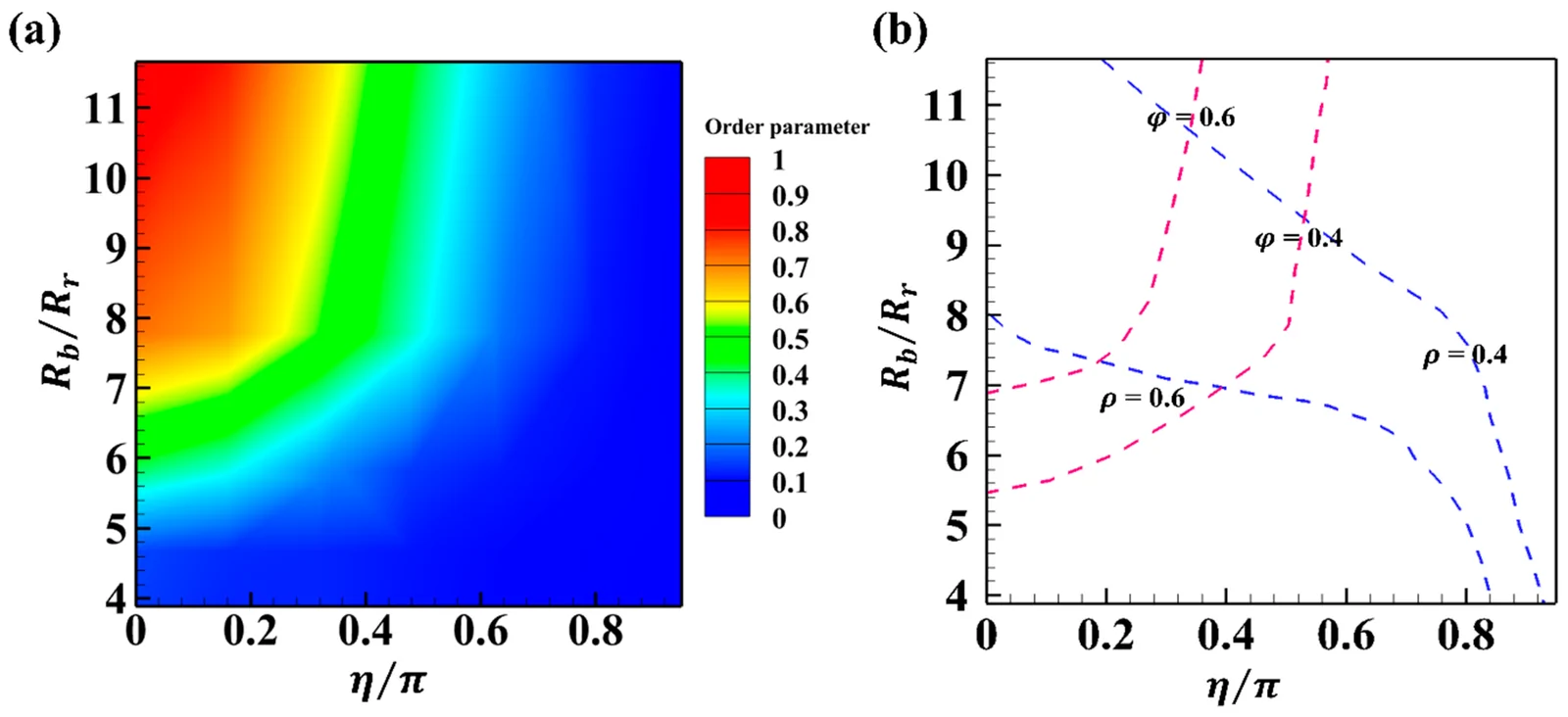
(**a**) Phase diagram of order transition (φ) as a function of normalised confinement $R_{b}$/$R_{r}$ and normalised noise strength (η/π). (**b**) Combined phase diagram of packing density and order parameter.

Figure 20a shows the order parameter φ as a function of $R_{b}/R_{r}$ and noise strength η/π, for systems with alignment interactions. The plot distinctly reveals three regions, each reflecting a different collective behaviour as governed by the interplay between confinement and stochasticity. The upper-left region of the plot, characterised by the darkest red hue, corresponds to the highest values of the order parameter. This region emerges for large $R_{b}/R_{r}$ and low η/π, signifying large boundary confinement and minimal noise strength, respectively. In this regime, particle alignment dominates over disorder, resulting in a robustly ordered phase with φ approaching unity. Transitioning toward the central region of the contour map, represented by green colouration, one observes a clear decline in φ. This region corresponds to either decreasing $R_{b}/R_{r}$ or increasing η/π from the red region, indicating that increasing confinement has a similar effect of increasing noise strength on deteriorating the global order φ, suggesting alignment competes with both the geometric restriction and the stochastic perturbation.

Finally, the third region, represented by the blue colour, corresponds to the lowest order parameter. In this region, η/π increases and $R_{b}/R_{r}$ decreases, leading to the lowest φ. In the upper-right region, both the $R_{b}/R_{r}$, and η/π are large. While the increase in the $R_{b}/R_{r}$ tends to increase φ, the high level of noise strength has a more significant disruptive effect. As a result, φ is lower in this region, showing that while the $R_{b}/R_{r}$, supports order, the noise strength overwhelms this effect and leads to a more disordered system. In the lower-right region, the boundary radius is small and η/π is relatively high. The order parameter remains low, indicating that the introduction of η/π similarly disrupts the order. In the lower-left region, where both the $R_{b}/R_{r}$, and η/π are small, φ is also relatively low. In this region, the system exhibits characteristics of a jammed state, where particles are more likely to prevent alignment even with minimal η/π. Figure 20b shows the combined phase diagram of packing density (blue lines) and order parameter (red lines). The regime defined by φ> 0.6 and ρ > 0.6 corresponds to a high-order and dense region, whereas the regime defined by φ> 0.4 and ρ > 0.4 represents an intermediate phase with moderate order and a dense region. The different regions provide guidance for designing an active particle system in a confined boundary with desired density and order parameter.

### 3.4. Effect of Interaction Radius R

**Figure 21:**
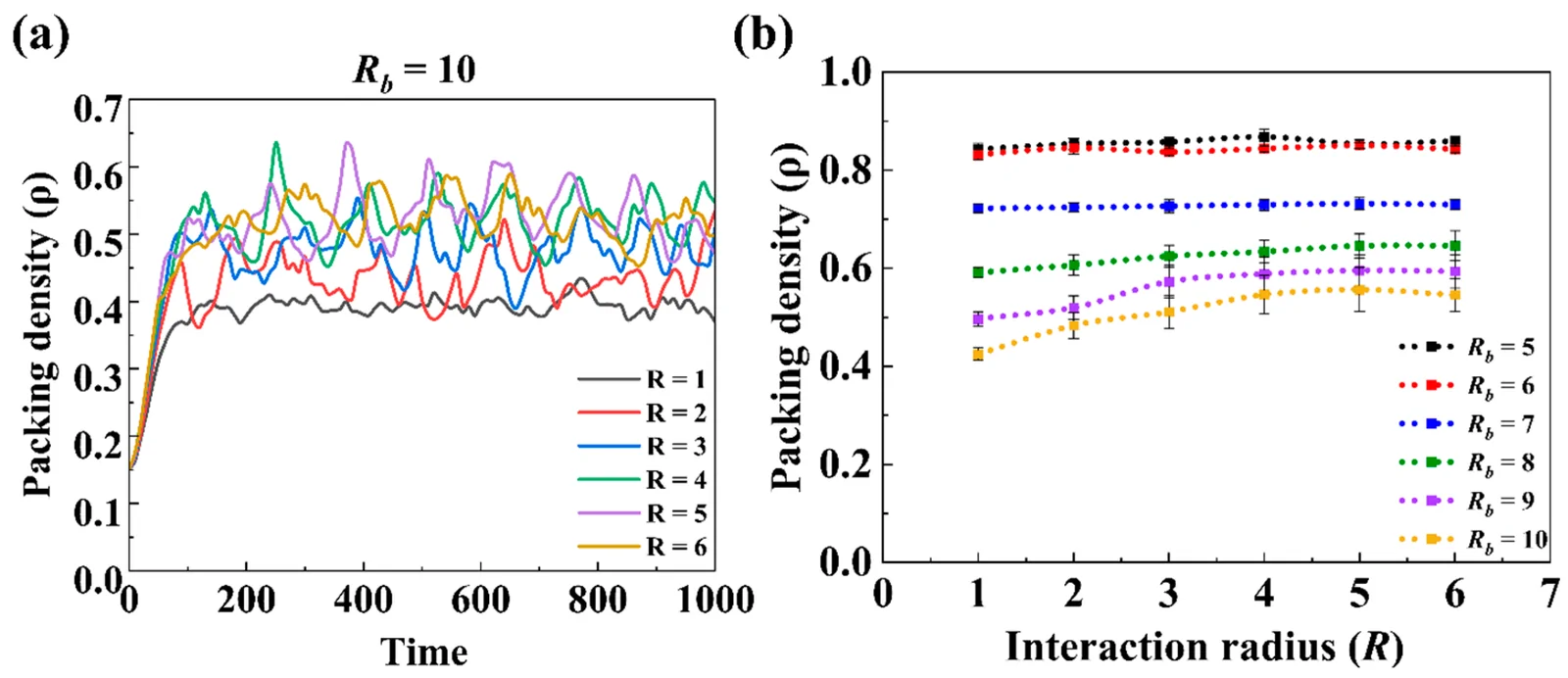
Effect of interaction radius (R) on packing density. (**a**) Time evolution of packing density under different interaction radii (R) with constant $R_{r}$ = 1.2, $R_{b}$ = 10 and η = 0.5. (**b**) Packing density (ρ) as a function of R for different $R_{b}$.

In the above analysis, the interaction radius *R* is fixed to be 6. The value is selected due to the fact that the effect of *R* on the packing behaviour of the considered system becomes saturated when *R* reaches 6, which is briefly discussed here. Firstly, Figure 21 shows the effect of *R* on packing density *ρ*. As shown in Figure 21a, under a typical condition of $R_{b}$ = 10, $R_{r}$ = 1.2 and *η* = 0.5, *ρ* shows a similar change with time, i.e., increasing sharply from 0 to about 100s, and then fluctuating around a constant value. The value of *ρ* at the steady state increases with *R* first but becomes similar when *R* is above 4. Figure 21b further shows the average packing density at the steady state as a function of *R* under different $R_{b}$, which further confirms that ρ for all $R_{b}$ is saturated at *R* = 6.

**Figure 22:**
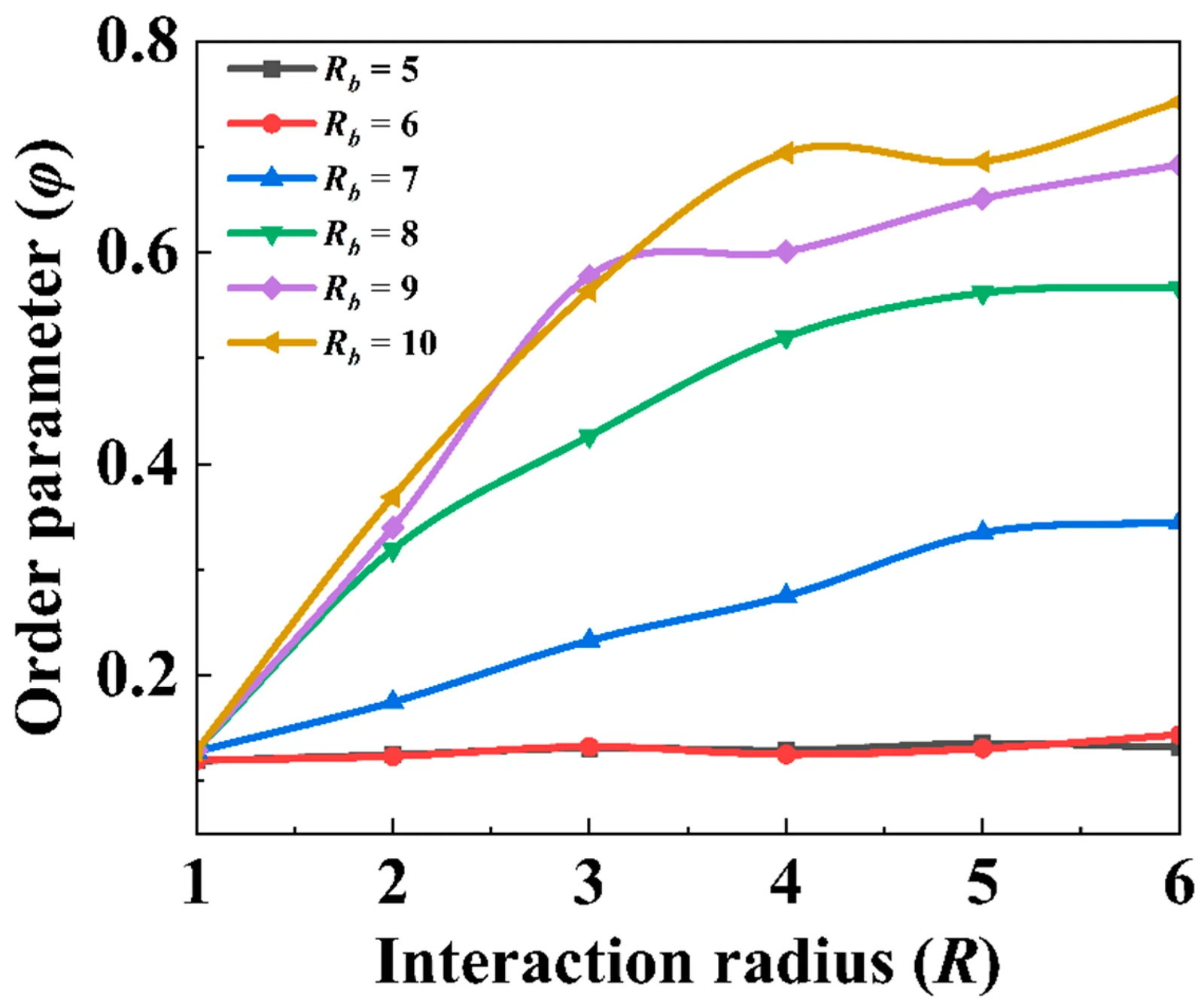
Effect of interaction radius (R) on the order parameter (φ) for different boundary radius ($R_{b}$) under $k_{a}$ = 1 with constant $R_{r}$ = 1.2 and η = 0.5.

In addition, the effect of interaction radius (R) on the order number φ has been investigated by varying *R* over a range of values, while keeping the noise amplitude fixed at η = 0.5. It can be seen that under different conditions, the order parameter increases sharply with R (see Figure 22). Beyond the interaction radius (*R* > 5), the order parameter saturates. Similar to the influence of packing density, increasing the interaction radius enhances the degree of collective alignment. Given that both ρ and φ become saturated when *R* increases to 6, *R* = 6 is used in our study.

## 4. Conclusions

In this work, we have investigated the effect of velocity alignment on the packing of active particles in a confined space, using a numerical model that integrates repulsion, alignment, noise strength, and boundary interactions. The effects of key variables on both the packing density and the polar order of the system have been studied by controlled numerical simulations. The packing structures of active particles have been systematically characterised using methods employed in granular packing studies. Based on the results, the key findings are summarised below.

In a small packing space (lower $R_{b}/R_{r}$), the system remains jammed, and the effect of the alignment interaction is suppressed. Consequently, the packing density (*ρ*) is not affected by the alignment interaction, and the polar order number (φ) is also jammed at a small value, even with the alignment interaction and low noise strength. As the packing space increases, the particles are unjammed, allowing the alignment interaction to play a more important role in increasing both *ρ* and φ. The phase diagram of packing density identifies a particular region where the alignment interaction increases packing density, which corresponds to relatively high $R_{b}/R_{r}$ and low noise strength. The packing structure of active particles is comparable to that of granular particles. The average coordination number increases with packing density. With the same CN, alignment makes particles require less space, acting as a micro-mechanism that enhances packing density. The local packing density exhibits a single-peak, symmetric distribution, and its standard deviation decreases with the global packing density. For active particles with an alignment interaction, the relationship between packing density and the polar order parameter shows a complicated change. When the packing space is large, with decreasing noise strength, increasing *ρ* is accompanied by an increase in φ. However, when the packing space starts to jam particles, increasing *ρ* is accompanied by a decrease in φ. In the combined packing density and order parameter phase diagram, a region with both moderate $R_{b}/R_{r}$ and moderate noise strength can yield relatively high order and dense packings. Interaction radius also influences both packing density and polar order number. Higher packing density and polar order number are achieved with a larger interaction radius.

Overall, our study reveals that the packing of active particles is affected by the interplay between confinement, “passive” interaction (repulsion), and “active” interaction (alignment). In particular, confinement sets the limits of motion, and alignment enables collective self-organisation once geometric constraints are relaxed. The work also bridges granular packing physics and active matter theory. By applying structural descriptors from passive systems, we show how active collectives transition between jammed, ordered, and disordered states. This framework not only deepens our understanding of non-equilibrium matter but also has practical implications for swarm robotics [64,65], including collective docking, return-to-base motion, area coverage, self-assembly, and congestion control [26,66,67], where packing and ordering influence efficiency, adaptability, and resilience. The predicted relationships among confinement, packing structure and collective order could be tested experimentally using Quincke rollers [68], active granular particles [69] or physical robot swarms. In these systems, the confinement size and particle number can be varied to control the global packing fraction, while activity or noise can be adjusted through the driving conditions. Physical robot swarms additionally allow the alignment rule and interaction range to be programmed directly, making them particularly suitable for testing the effects of alignment considered in the present model. Measurements of global polar order, local Voronoi density, and coordination-number distributions would permit direct comparison with the quantities analysed here. Further extensions incorporating adaptive or learning-based interaction rules may reveal richer collective phenomena, while integration with machine learning and real-time sensing could enable smart swarms capable of autonomously optimising their collective structure for diverse tasks.

## Figures and Tables

**Table 1:** Parameters used in simulations.

Definition, Parameters, Unit	Value
Number of particles, *N*, Dimensionless	100
Length of the domain in *x*-direction, $L_{x}$, SLU	25
Length of the domain in *y*-direction, $L_{y}$, SLU	25
Radius of the circular boundary, $R_{b}$, SLU	5–10
Speed of particles, v, SLU/STU	0.1
Noise strength, η, rad	0 to 3
Radius of interaction for alignment, *R*, SLU	1–6
Repulsion strength $k_{r}$, Dimensionless	10^6^
Alignment strength $k_{a}$, Dimensionless	0 and 1
Boundary strength $k_{b}$, Dimensionless	3
Radius of repulsion, $R_{r}$, SLU	0.6–1.8
Time step size, *dt*, STU	1
Number of simulation steps, *T*, Dimensionless	1000–5000

## Data Availability

The data supporting the findings of this study are available from the corresponding author upon reasonable request.

## Supplementary Materials

The following supporting information can be downloaded at: https://www.mdpi.com/article/10.3390/mi17080884/s1, Figure S1: Mean heterogeneity index versus boundary radius; Figure S2: Mean heterogeneity index versus repulsion radius; Figure S3: Mean heterogeneity index versus noise strength; Table S1: Local density heterogeneity for the boundary radius; Table S2: Local density heterogeneity for the repulsion radius; Table S3: Local density heterogeneity for the noise strength. Video S1: Particle packing simulation ($R_{b}\;$ = 7, $k_{a}$ = 0); Video S2: Particle packing simulation ($R_{b}$ = 7, $k_{a}$ = 1); Video S3: Particle packing simulation ($R_{b}$ = 10, $k_{a}$ = 0); Video S4: Particle packing simulation ($R_{b}$ = 10, $k_{a}$ = 1).

## Abbreviations

The following abbreviations are used in this manuscript:

N	Number of particles
$L_{x}$	Length of the domain in the x-direction
$L_{y}$	Length of the domain in the y-direction
$R_{b}$	Radius of the circular boundary
$R_{r}$	Radius of repulsion
*R*	Radius of interaction
v	Speed of particles
ε	Noise, a random number uniformly distributed within $\left[-\eta ,\eta \right]$
η	Noise strength
$k_{r}$	Repulsion strength
$k_{a}$	Alignment strength
$k_{b}$	Boundary strength
dt	Time step
t	Timesteps
T	Total number of time steps
$\theta_{i}$	Moving direction vector of the particle i
$\theta_{j}$	Moving direction vector of the particle j
$\theta_{r,i}$	Velocity direction resulting from the repulsion interaction
$\theta_{a,i}$	The velocity direction resulting from the alignment interaction
${\hat{d}}_{ij}$	Direction vector from particle i to particle j
$d_{ij}$	Distance between particles i and particle j
$N_{a}$	Set of neighbouring particles for alignment
$N_{r}$	Set of neighbouring particles for repulsion
${\hat{d}}_{i,c}$	Velocity direction towards the inside of the boundary
$d_{i,R_{b}}$	Distance between the particle i and $R_{b}$
${\hat{d}}_{i,t}$	Direction vector to the target point
*SLU*	Simulation length unit
*STU*	Simulation time unit
ρ	Global packing density
$\rho_{local}$	Local packing density
$A_{Vor,i}$	Area of the Voronoi cell
$A_{i}$	Area of the particle
$N_{int}$	Total number of fully enclosed internal Voronoi cells
CN	Coordination number
φ	Order number
