# Supplementary material for: Effect of Velocity Alignment on the Packing of Active Particles
Source: Micromachines (Basel). 2026 Jul 24;17(8):884. doi: 10.3390/mi17080884 (PMC13515317; doi:10.3390/mi17080884)
Supplement: Supplementary file 1 [file micromachines-17-00884-s001.zip › micromachines-4401386-supplementary.pdf]

Local Density Heterogeneity Analysis

The local-density heterogeneity index was calculated $H(t) = \frac{\sigma_\rho}{\bar{\rho}(t)}$, where $\bar{\rho}(t)$ and σ_ρ are the mean and standard deviation of the local densities

1. Effect of boundary radius

Table S1. Local density heterogeneity for the boundary radius.

Rb	Mean H (Ka=0)	H SD (Ka=0)	Mean H (Ka=1)	H SD (Ka=1)	Difference (Ka1−Ka0)	% change with alignment
5	0.0595	0.0081	0.0574	0.0074	-0.0021	-3.5%
6	0.0618	0.0077	0.0573	0.0068	-0.0045	-7.3%
7	0.0947	0.0107	0.0896	0.0097	-0.0052	-5.5%
8	0.1504	0.0166	0.1257	0.0160	-0.0247	-16.5%
9	0.2073	0.0198	0.1542	0.0211	-0.0532	-25.6%
10	0.2619	0.0263	0.1740	0.0204	-0.0879	-33.6%

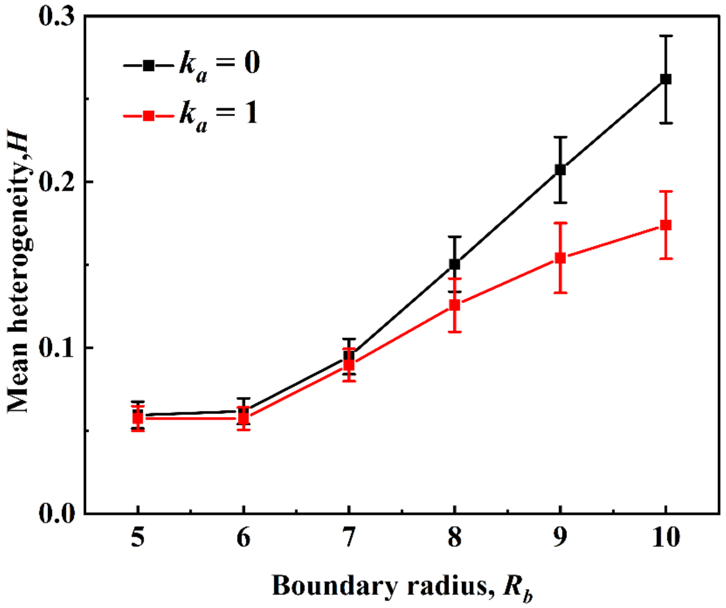

Figure S1. Mean heterogeneity index versus boundary radius. Error bars show the temporal standard deviation of $H(t)$.

2. Effect of repulsion radius

Table S2. Local density heterogeneity for the repulsion radius.

Rr	Mean H (Ka=0)	H SD (Ka=0)	Mean H (Ka=1)	H SD (Ka=1)	Difference (Ka1−Ka0)	% change with alignment
0.6	0.4285	0.0453	0.2704	0.0331	-0.1581	-36.9%
0.9	0.2369	0.0231	0.1756	0.0253	-0.0613	-25.9%
1.2	0.0954	0.0108	0.0902	0.0098	-0.0052	-5.5%
1.5	0.0543	0.0065	0.0551	0.0061	0.0007	1.4%
1.8	0.0443	0.0072	0.0483	0.0064	0.0040	9.1%

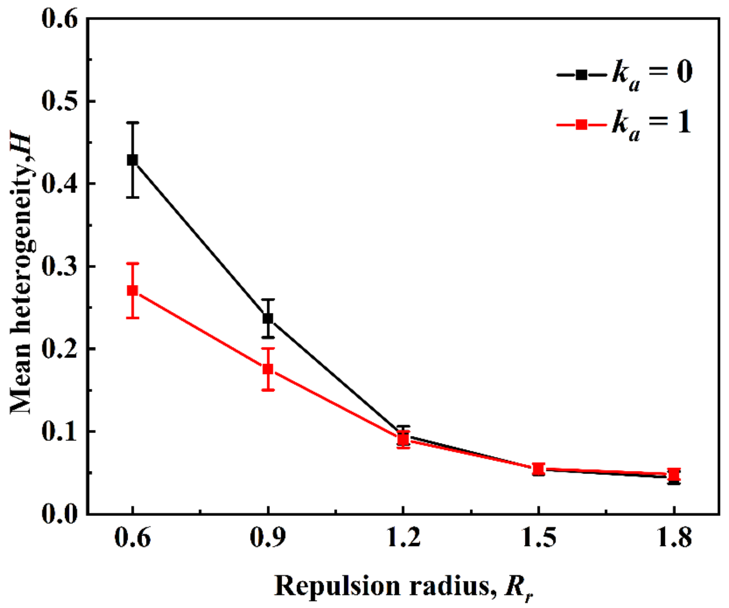

Figure S2. Mean heterogeneity index versus repulsion radius. Error bars show the temporal standard deviation of $H(t)$.

3. Effect of noise strength

Table S3. Local density heterogeneity for the noise strength.

Noise strength	Mean H (Ka=0)	H SD (Ka=0)	Mean H (Ka=1)	H SD (Ka=1)	Difference (Ka1−Ka0)	% change with alignment
0	0.2633	0.0248	0.1563	0.0216	-0.1070	-40.6%
0.5	0.2638	0.0265	0.1753	0.0205	-0.0885	-33.6%
1	0.2654	0.0239	0.2268	0.0289	-0.0386	-14.5%
1.5	0.2525	0.0297	0.2306	0.0264	-0.0219	-8.7%
2	0.2877	0.0416	0.2634	0.0297	-0.0243	-8.5%
2.5	0.4339	0.0412	0.3866	0.0608	-0.0473	-10.9%

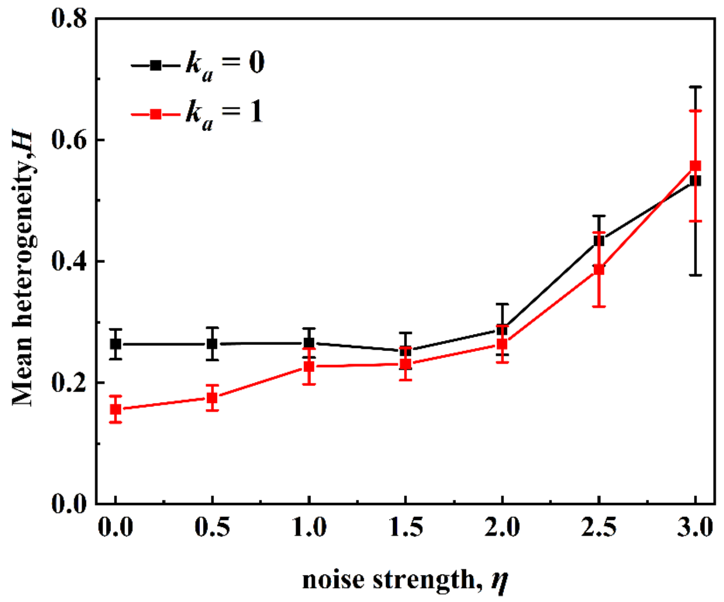

Figure S3. Mean heterogeneity index versus noise strength. Error bars show the temporal standard deviation of $H(t)$.
